# Seasonal dynamics of diet–gut microbiota interaction in adaptation of yaks to life at high altitude

**DOI:** 10.1038/s41522-021-00207-6

**Published:** 2021-04-20

**Authors:** Na Guo, Qunfu Wu, Fuyu Shi, Jiahuan Niu, Tao Zhang, A. Allan Degen, Qiangen Fang, Luming Ding, Zhanhuan Shang, Zhigang Zhang, Ruijun Long

**Affiliations:** 1 School of Life Science, State Key Laboratory of Grassland Agro-ecosystems, Lanzhou University, Lanzhou, Gansu China; 2 State Key Laboratory for Conservation and Utilization of Bio-Resources in Yunnan, School of Life Sciences, Yunnan University, Kunming, Yunnan China; 3 Desert Animal Adaptations and Husbandry, Wyler Department of Dryland Agriculture, Blaustein Institutes for Desert Research, Ben-Gurion University of the Negev, Beer Sheva, Israel; 4 College of Grassland Science/Key Laboratory of Grassland Ecosystem of the Ministry of Education, Gansu Agricultural University, Lanzhou, Gansu China; 5 State Key Laboratory of Genetic Resources and Evolution, Laboratory of Evolutionary & Functional Genomics, Kunming Institute of Zoology, Chinese Academy of Sciences, Kunming, Yunnan China

**Keywords:** Microbiome, Microbial ecology

## Abstract

Dietary selection and intake affect the survival and health of mammals under extreme environmental conditions. It has been suggested that dietary composition is a key driver of gut microbiota variation; however, how gut microbiota respond to seasonal dietary changes under extreme natural conditions remains poorly understood. Sequencing plant *trn*L (UAA) region and 16S rRNA gene analysis were employed to determine dietary composition and gut microbiota in freely grazing yaks on the Tibetan plateau. Dietary composition was more diverse in winter than in summer, while Gramineae and Rosaceae were consumed frequently all year. Turnover of seasonal diet and gut microbiota composition occurred consistently. Yaks shifted enterotypes in response to dietary change between warm and cold seasons to best utilize nitrogen and energy, in particular in the harsh cold season. Our findings provide insights into understanding seasonal changes of diet–microbiota linkages in the adaptation of mammals to high altitudes.

## Introduction

The Tibetan plateau, called ‘the third pole’, forms the largest and highest year-round grazing area in the world. The region is characterized by a harsh climate of extreme cold and aridity, and high ultraviolet radiation and hypoxia, which challenge the survival of humans and other mammals^1^. The yak (*Bos grunniens*), an iconic symbol of high altitude and a mainstay for Tibetan people^2^, has anatomical and physiological adaptations and a genetic basis for mammalian adaptations^3^, as well as a co-evolved microbiome^4^, that equip the animal for the high altitude and extreme environment. Yet gut microbiota of the yak and their relationship to seasonal dietary shifts in their natural habitat remain largely unknown, although this information could contribute to the understanding of adaptation to the high-altitude Tibetan plateau.

Gut microbiota are complex and dynamic^5^, being sensitive to perturbations, such as dietary changes, environmental factors^6^ and enteric pathogens. They play an integral role in nutrient intake, behavior, metabolism, immune function, and development of the host^7,8^. Substantial changes in mammalian microbiota composition have been observed in response to seasonal diet availability among and within individuals, as evidenced by longitudinal analyses of gut microbiota in Hadza hunter-gatherers^9^, wild wood mice (*Apodemus sylvaticus*)^10^, red squirrels (*Tamiasciurus hudsonicus*)^6^, giant pandas (*Ailuropoda melanoleuca*)^11^, wild great apes^12,13^ and North American bison (*Bison bison*)^14^. However, most studies on mammalian gut microbiota dynamics were done in non-stressful environments and without quantitative dietary information related to habitats. Recent reports on gut microbiota composition from large herbivores in the semi-arid East African savanna revealed a greater seasonal turnover and diet–microbiota association in domesticated than in wild species^15^. These studies provide a better understanding of intra-specific and inter-specific diet–microbiota associations in wild and domesticated species. Yet studies on seasonal diet and microbiota relations are lacking in high-altitude mammals.

To examine the fine-scale relationship between quantitative dietary consumption and gut microbiota, we conducted a spatio-temporal study of the impact of seasonal diet on gut microbiota from 302 individual yaks. Using DNA metabarcoding and 16S rRNA gene analyses, measurements were made in free-grazing yaks across four seasons on the eastern part of the Tibetan plateau, in which either a transhumance (TH) or open-continuous grazing (OCG) regime was followed. These in-depth and longitudinal analyses could contribute to the understanding of the adaptations of mammals to the harsh, high altitude environment.

## Results

### Established reference database for DNA metabarcoding analysis

According to the National Center for Biotechnology Information (NCBI) and Bold system databases, there were 199 plant species in the study areas. Combined with plant taxonomic identification, this study generated an additional 212 local plant species DNA barcode reference library using P6 loop of the chloroplast *trn*L (UAA) intron marker genes^16^ (see the “Methods” section). This revised library was used for DNA metabarcoding analysis. In total, our library comprised 411 plant species (included all species from the sampling region; local plant list recorded 386 species^17^) throughout the alpine grassland area of the study in the TH and OCG regimes.

### Diet diversity and composition across seasons

**Fig. 1:**
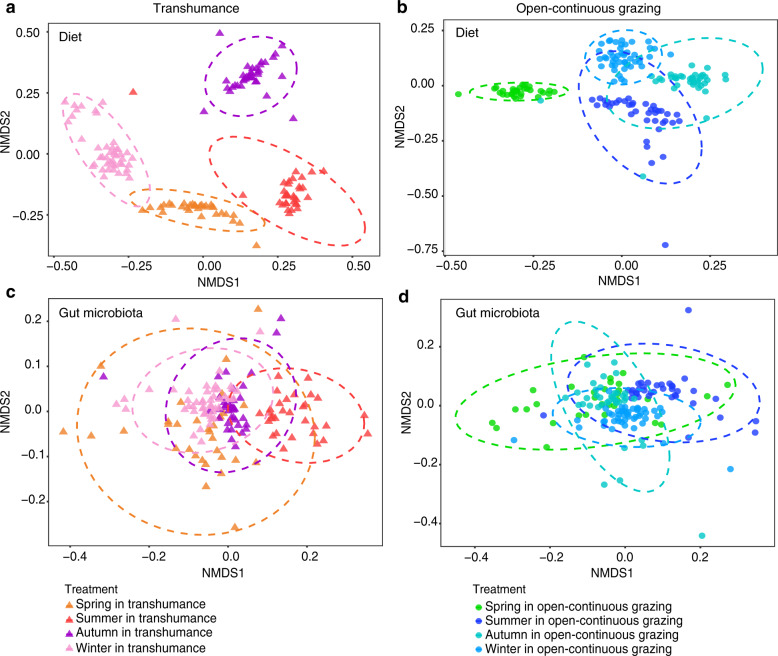
Seasonal changes in both diet and gut microbiota community structures of yaks in transhumance and open-continuous grazing regimes. Within and among seasons, Bray–Curtis dissimilarity in diet and microbiota are presented in Supplementary Table 2. Rows show the same ordinations for diet (**a** and **b**) and microbiota (**c** and **d**) compositions. Diet composition and gut microbiota represent transhumance (**a** and **c**) and open-continuous grazing (**b** and **d**) regimes. Individual yak diet compositions from samples collected in (**a**) spring (*n* = 32), summer (*n* = 33), autumn (*n* = 37), and winter (*n* = 45) in transhumance grassland (anosim analysis: *R* = 0.94, *p* = 0.0001; adonis analysis: *R*^2^ = 0.78, *p* = 0.0001), (**b**) spring (*n* = 31), summer (*n* = 39), autumn (*n* = 38), and winter (*n* = 47) in open-continuous grazing grassland (anosim analysis: *R* = 0.88, *p* = 0.0001; adonis analysis: *R*^2^ = 0.67, *p* < 0.0001), and gut microbiota compositions in (**c**) spring (*n* = 31), summer (*n* = 31), autumn (*n* = 37) and winter (*n* = 48) in transhumance grassland (anosim analysis: *R* = 0.50, *p* < 0.0001; adonis analysis: *R*^2^ = 0.16, *p* < 0.0001) and (**d**) spring (*n* = 31), summer (*n* = 37), autumn (*n* = 38), winter (*n* = 47) in open-continuous grazing grassland (anosim analysis: *R* = 0.47, *p* < 0.0001; adonis analysis: *R*^2^ = 0.16, *p* < 0.0001) plotted on nonmetric multidimensional scaling (NMDS) according to the Bray–Curtis dissimilarity. Analysis of similarities (ANOSIM), adonis analysis and permutational multivariate analysis of variance (PERMANOVA) were used for statistical testing of treatment similarities. The dotted ellipse borders represent the 95% confidence interval.

**Fig. 2:**
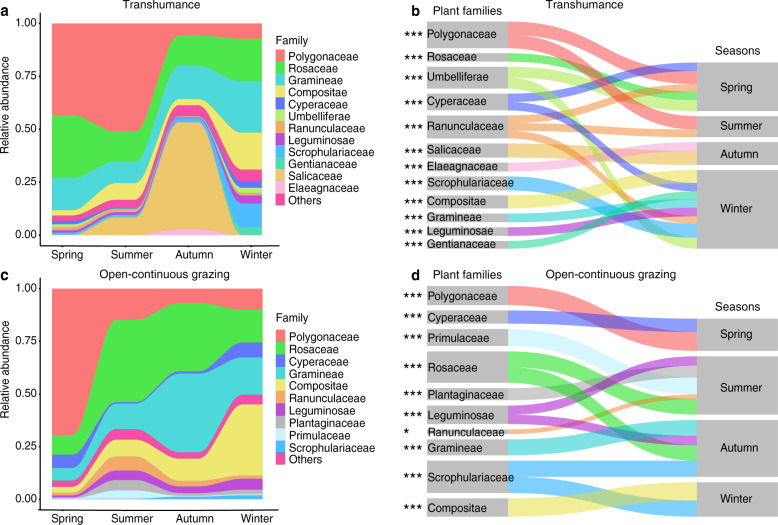
Seasonal changes of dietary compositions of yaks in transhumance and open-continuous grazing regimes. Stream-graph displays the relative abundance of plant family-level taxa in spring, summer, autumn, and winter in transhumance (**a**) and open-continuous grazing (**c**) regimes. Low abundance taxa (<5%) are grouped together as “others”. Indicator families that are related to each season are tracked using Sankey plots in transhumance (**b**) and open-continuous grazing (**d**) regimes. Lines represent associations between indicator families and seasons, which are colored by plant family. Line width is scaled to reflect indicator value (higher indicator value of family is more strongly associated with season). Indicator values are presented in Supplementary Fig. 7. The statistical *p* values mean the family associated with seasons, *p < 0.05, **p < 0.01, ***p < 0.001.

The diet data included 30,534,414 high-quality sequences, 81,530 unique sequences after removal of singletons and 2010 operational taxonomic units (OTUs) (Supplementary Table 3). All dietary sequences represented 41 plant families, 83 genera, and 80 species. Dietary composition was more diverse in winter than in summer in both grazing regimes and displayed an evident separation seasonally (Fig. 1a, b and Supplementary Figs. 4, 5b, e). Gramineae and Rosaceae were consumed by yaks frequently throughout the year in both TH and OCG grasslands (Fig. 2a, c). The highest relative abundances were Polygonaceae, Rosaceae, and Gramineae in spring and summer, and Gramineae, Rosaceae, and Compositae in winter in both TH and OCG grasslands. In autumn, Gramineae and Rosaceae were highest in both TH and OCG grasslands, and Salicaceae was also high in TH. As indicator plant species, Polygonaceae was identified in spring, Scrophulariaceae and Compositae in winter in both TH and OCG regimes, Polygonaceae in TH and Rosaceae in OCG in summer and Salicaceae in TH and Gramineae in OCG in autumn (Fig. 2b, d; Supplementary Fig. 7a, b). The distinct seasonal diets provided a sound basis to identify the effects of seasonal diet patterns on yak gut microbiota.

**Fig. 3:**
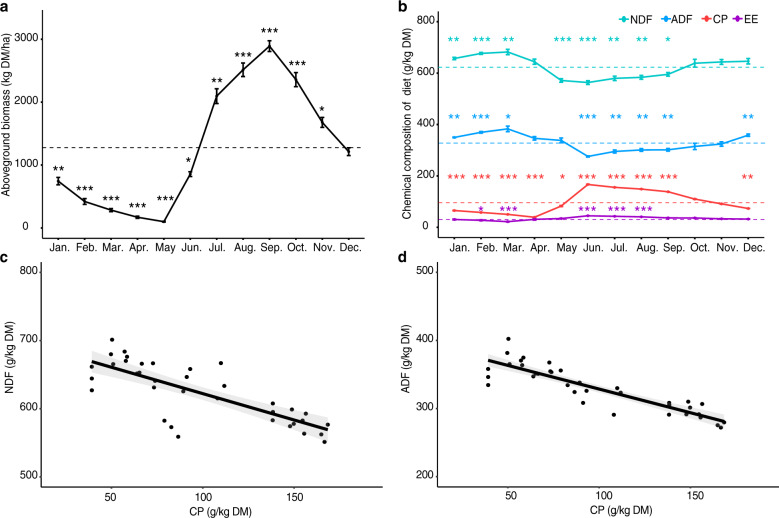
Seasonal dynamics in above-ground biomass (AGB) and chemical composition (dry matter basis) of the diets year-round in the open-continuous grazing grassland. **a** Line chart represents the AGB (kg DM/ha). The dashed line is the mean AGB year-round. **b** Line chart represents chemical composition (crude protein (CP), ether extract (EE), acid detergent fiber (NDF), and neutral detergent fiber (ADF)) of diet year-round. The dashed lines are the mean of each chemical composition year-round and are colored by each chemical composition. Values and error bars are shown as means ± SE. Average contents of CP are correlated negatively with those of NDF (*R*^2^ = 0.59, *p* < 0.001) (**c**) and ADF (*R*^2^ = 0.78, *p* < 0.001) (**d**). Statistic tests are performed by the *t*-test with FDR (false discovery rate) corrected *p*-value, *p < 0.05, **p < 0.01, ***p < 0.001.

In the OCG regime, above-ground biomass (AGB) was highest in the warm season (from June to September), with 2891 ± 148 kg DM/ha in September, and was lowest in the cold season (from October to May) with 99 ± 10.8 kg DM/ha in May (Fig. 3a). Neutral detergent fiber (NDF) and acid detergent fiber (ADF) were higher by 10% and 16%, respectively, while crude protein (CP) and ether extract (EE) were lower by 53% and 25%, respectively, in the cold than the warm season (Fig. 3b). Specifically, the highest CP (166 ± 10.2 g/kg DM) occurred in June, and the lowest (39 ± 5.5 g/kg DM) in April. Similar trends were observed in the TH regime (Supplementary Fig. 3a, b). Consequently, yaks were forced to cope with sparse forage of low-nitrogen content in the cold season.

### Gut microbiota diversity and composition across seasons

**Fig. 4:**
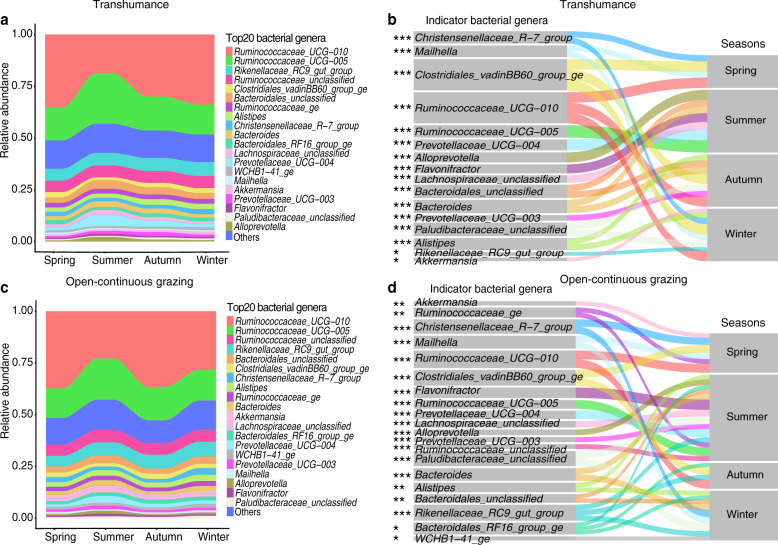
Seasonal variations of yak gut microbiota at the genus level in transhumance and open-continuous grazing regimes. Relative abundance of the 20 most abundant genera over seasons (spring, summer, autumn, and winter) are aggregated and colored on a stream-graph in (**a**) transhumance and (**c**) open-continuous grazing regimes. Low abundance taxa (except for 20 most abundant genera) are grouped together as “others”. Indicator genera that are related to each season are tracked using Sankey plots in transhumance (**b**) and open-continuous grazing (**d**) regimes. Lines represent associations between indicator genera and seasons, which are colored by genus level. Line width is scaled to reflect indicator value (higher indicator value of genus is more strongly associated with the season). Indicator value are shown in Supplementary Fig. 7. The statistical *p* values mean the genus associated with seasons. *p < 0.05, **p < 0.01, ***p < 0.001.

The gut microbiota included 25,375,771 high-quality sequences, 1,125,235 unique sequences after removal of singletons and 14,239 OTUs (Supplementary Table 3). Seasonal shifts in gut microbiota were evident in both the TH and OCG regimes (Fig. 1c, d). In total, 18 gut bacteria phyla were identified, with Firmicutes and Bacteroidetes the most abundant, regardless of season and grazing regime (Supplementary Fig. 6, Supplementary Fig. 7c, d). Distinct seasonal dynamics were exhibited at the genus-level in both TH and OCG grasslands, with *Ruminococcaceae_UCG-010*, *Ruminococcaceae_UCG-005*, *Rikenellaceae_RC9_gut_group*, unclassified *Ruminococcaceae* and unclassified *Bacteroidales* the most abundant across seasons (Fig. 4a, c). Moreover, the relative abundance of *Prevotellaceae_UCG-004* increased during the summer. Indicators at the genus-level displayed seasonal and spatial differences. In the TH regime, in spring, autumn, and winter, the indicator species were *Ruminococcaceae_UCG-010* and *Clostridiales_vadinBB60_group*; whereas, in summer, they were *Ruminococcaceae_UCG-005* and *Prevotellaceae_UCG-004*. In the OCG regime, in spring and summer, the indicator species were *Ruminococcaceae_UCG-010* and *Clostridiales_vadinBB60_group*; in autumn, they were *Flavonifractor*, *Ruminococcaceae_UCG-005*, and *Prevotellaceae_UCG-004*; whereas, in winter it was the unclassified *Paludibacteraceae* (Fig. 4b, d; Supplementary Fig. 7e, f).

### Diet associated with overall microbiota composition across seasons

**Fig. 5:**
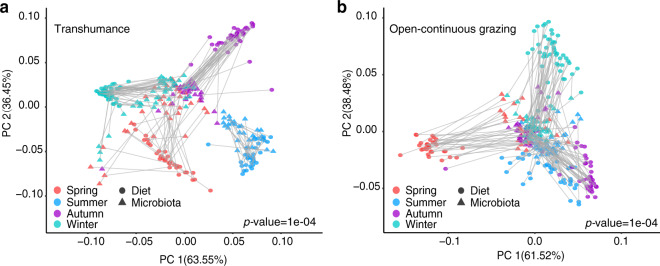
Seasonal clustering patterns in diet–microbiota lineages of yak in transhumance and open-continuous grazing regimes. The diet and microbiota compositions are determined with Bray–Curtis distance in (**a**) transhumance and (**b**) open-continuous grazing regimes, which are colored by season. Procrustes rotates the results of separate principal coordinates of diet composition (circle symbols) and gut microbiota composition (triangle symbols). Significant dissimilarities occurred between diet and microbiota ordinations by *p*-value.

We applied Procrustes analysis to test diet and microbiota variations across seasons. When analyzed using Bray–Curtis (BC) dissimilarities, seasonal diet composition was associated with microbiota composition consistently in both TH (*p* = 0.0001, Fig. 5a) and OCG regimes (*p* = 0.0001, Fig. 5b). However, microbiota richness was not correlated with dietary richness (Supplementary Fig. 8).

**Fig. 6:**
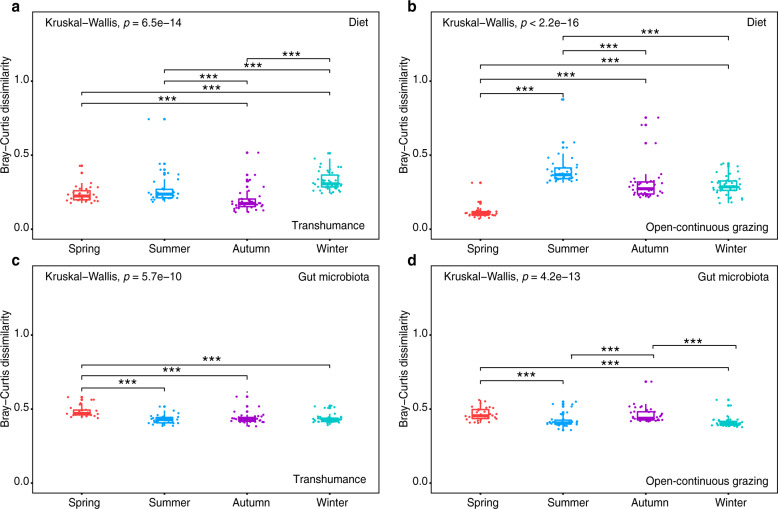
Bray–Curtis dissimilarities within seasons in dietary diversity and gut microbial diversity across seasons in transhumance and open-continuous grazing regimes. Rows show the dissimilarities for diet (**a** and **b**) and gut microbiota (**c** and **d**) within seasons. Each column organizes the data so that diet and gut microbiota Bray–Curtis dissimilarities represent transhumance (**a**, **c**) and open-continuous grazing (**b**, **d**) regimes. All boxplot distributions are tested by non-parametric Kruskal–Wallis and Wilcoxon with FDR (false discovery rate) corrected *p*-value, center values indicate the median and error bars. *p < 0.05, **p < 0.01, ***p < 0.001.

Diet and microbiota dissimilarities were consistently higher among than within seasons (Fig. 6 and Supplementary Table 2). Diet separations across seasons were smallest between spring and summer (0.482) in TH and between autumn and winter (0.566) in OCG (Supplementary Table 2), and were greatest between summer and winter (0.779) in TH and between spring and autumn (0.734) in OCG, suggesting that consumption of plant species occurred according to availability (Supplementary Table 2). Most overlapping microbiota occurred between autumn and winter while most non-overlapping microbiota occurred between spring and summer in both TH and OCG, which indicated that microbiota remained relatively stable (Supplementary Table 2). Within season, there was a larger difference in diet dissimilarity (0.117–0.385) than in microbiota dissimilarity (0.432–0.486) in the two grazing regimes, suggesting that stable gut microbiota communities may contribute to host adaptation to the extreme environment (Fig. 6 and Supplementary Table 2).

### Gut enterotypes and functional context represented by *Akkermansia* and uncultured *Eubacterium WCHB1-41* for underlying cold adaptation

**Fig. 7:**
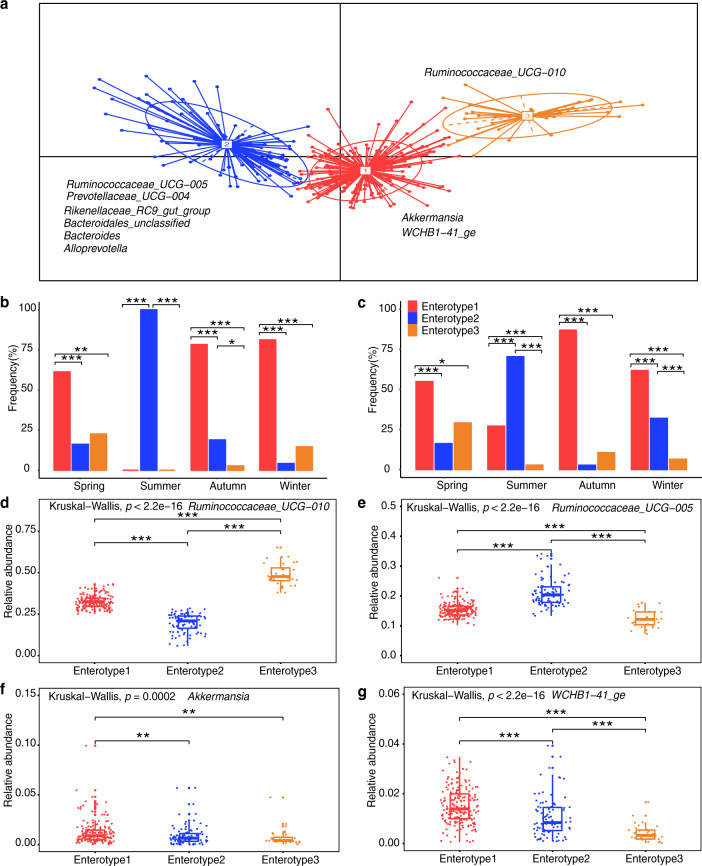
Enterotype distributions of yak gut microbiota associated with season using Bray–Curtis dissimilarity. Identification of yak enterotypes is presented in Supplementary Fig. 9. **a** Visualizations of enterotypes, as identified by PAM (partitioning around medoid) clustering. Genera corresponding to each enterotype are identified by their relative abundance (see Supplementary Fig. 10). **b** and **c** Proportion of samples for each enterotype in spring, summer, autumn, and winter in (**b**) transhumance and (**c**) open-continuous grazing regimes. **d**–**g** Relative abundance of bacterial taxa characteristic of each enterotype. Ten genera were chosen based on their average contribution to overall Bray–Curtis dissimilarity. All six bacterial genera are presented in Supplementary Fig. 10. Colors correspond to enterotype clusters. All bar distributions are tested by Fisher’s exact test with FDR (false discovery rate) corrected two-tailed *p*-values (**b** and **c**). All boxplot distributions are tested by non-parametric Kruskal–Wallis and Wilcoxon with FDR-corrected *p*-value, center values indicated the median and error bars (**d**–**g**). *p < 0.05, **p < 0.01, ***p < 0.001.

Based on the report that enterotypes exhibit functional differences^18^, we examined whether yak gut microbiota partitioned into clusters that differ in functional properties according to seasonal dietary intake. Principal component analysis (PCA) revealed that the samples formed three distinct enterotype clusters based on BC dissimilarities. Each cluster was driven by the variation of its representative genera level: *Akkermansia* and uncultured *Eubacterium WCHB1-41* in Enterotype 1, *Ruminococcaceae_UCG-005* in Enterotype 2 and *Ruminococcaceae_UCG-010* in Enterotype 3 (Fig. 7a, d–g; Supplementary Figs. 9 and 10). In TH and OCG, there was a change in enterotype assignment across seasons. Enterotype 1 occurred predominantly in the cold season (spring, autumn, and winter), Enterotype 2 in the warm season (summer) (Fig. 7b, c; *p* < 0.05, Fisher’s exact test) and Enterotype 3 was prevalent throughout the year (Fig. 7b, c). This study identifies the distribution of different enterotypes across seasons in high altitude yaks.

**Fig. 8:**
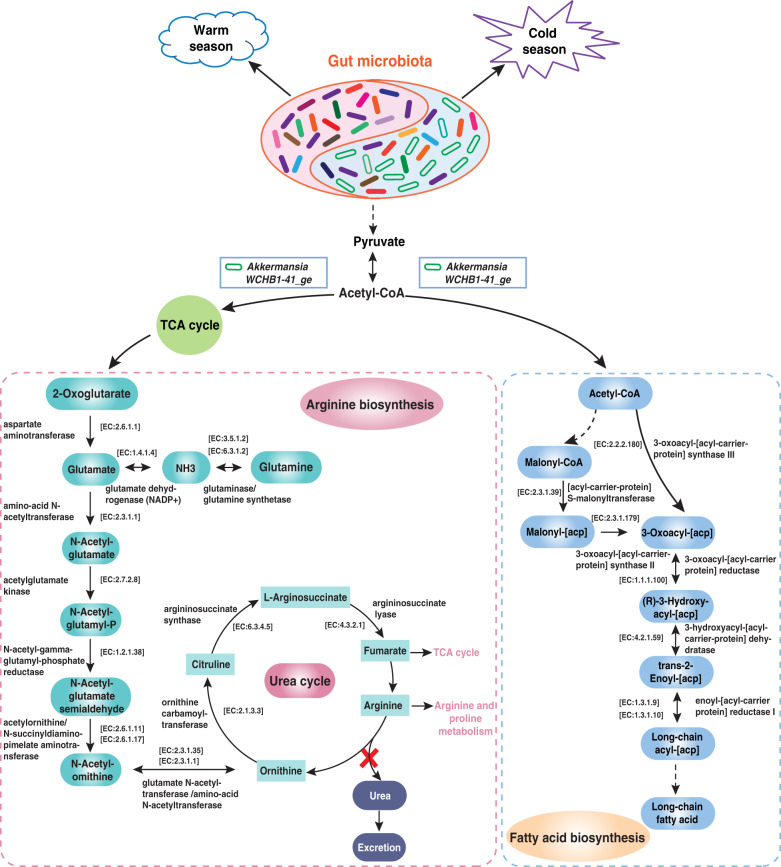
Metabolic pathway associated with Akkermansis and uncultured *Eubacterium* WCHB1-41_ge on host metabolism. The compositions of Akkermansis and uncultured *Eubacterium* WCHB1-41_ge are the key factors that influence arginine biosynthesis and fatty acid biosynthesis and were significantly higher during the cold season. These microbiota may contribute to energy production during cold season, which is the period of insufficient dietary and protein intake. All enzymes and EC (Enzyme Nomenclature) numbers were obtained from Kyoto Encyclopedia of Genes and Genomes (KEGG) database.

These seasonal distributions of gut enterotypes led us to hypothesize that the fixed gut enterotype, represented by *Akkermansia* and uncultured *Eubacterium WCHB1-41*, plays a vital role in regulating nutritional requirements in the cold season with sparse forage. To test this hypothesis, we examined the functional relevance of *Akkermansia* and Kiritimatiellaeota based on genus-level pan-genomes in the Kyoto Encyclopedia of Genes and Genomes (KEGG) database. Both *Akkermansia* and Kiritimatiellaeota showed convergent enrichment of enzymes that are involved in arginine and fatty acid biosynthesis pathways (map00220 and map00061) (Fig. 8). Pyruvate generated acetyl-CoA, mediated by both *Akkermansia* and Kiritimatiellaeota, enters the tri-carboxylic acid (TCA) cycle and involves arginine biosynthesis, and also regulates fatty acid synthesis. Notably, we observed 12 enzymes that play key roles in arginine biosynthesis, but no enzyme is involved in urea synthesis to reduce nitrogen loss in urine under conditions of low-nitrogen stress. Six of a total of seven enzymes participated directly in fatty acids biosynthesis and contributed to energy deposition. These results indicated that both arginine and fatty acid biosynthesis pathways evolved in high altitude mammals for efficient nitrogen utilization and energy deposition in the cold season.

## Discussion

We determined seasonal shifts in diet and gut microbiota in a high-altitude large herbivore. Yaks graze the grasslands of the Tibetan plateau all year, and face severe challenges, in particular extreme cold and limited food availability during the cold season. To survive under such conditions, yaks have evolved anatomical and physiological adaptations. Furthermore, convergent evolution was reported for yaks and their rumen microbiota. The rumen microbiota follow unique maturation strategies^19^ and differ in composition from microbiota of lowland ruminants^4^.

In this study, seasons constrained both diet and gut microbiota, but less so for microbiota than diet. Such effect of season on microbiota composition in response to food availability and chemical composition might be due to a re-assembly of the community structure of the gut microbiota resulting from substrate availability. Surprisingly, among seasons, the changes in gut microbiota composition were relatively stable compared with changes in dietary composition, which suggests that high altitude mammals evolved stable patterns of gut microbiota composition across seasons. By contrast, gut microbiota in humans and other mammals differed markedly with seasonal dietary shifts^9,10^. In response to cyclic seasonal dietary fluctuations of the host, gut microbiota could alter their metabolic rate and energy extraction from complex carbohydrates, and, ultimately, promote the co-evolution of hosts and microbes^6^. Furthermore, our findings support the present grassland polices of TH of moving yaks to different landscapes and searching for favorable diet. This practice mitigates grazing pressure, thereby enhancing animal performance. As demonstrated by our diet and microbiota analyses, a better understanding of relations between yaks and plants can yield key insights in supporting TH with a proper stocking rate and improving grassland management strategies. The key microbiota associated with feed efficiency could provide an alternative solution for strengthening microbiota-led breeding programs to increase yak performance, particularly in the cold season.

The lack of precise methods to assess and identify the complex diet consumed by grazing herbivorous mammals has been a long-standing obstacle in field studies. Conventional methods include field observations, microhistology and natural n-alkanes from plant cuticular waxes^20,21^. However, these methods are not suitable for diet analyses of herbivorous mammals feeding on pasture with high plant diversity. Field observations require high visibility and are prone to omission of plant species^22^, while histology is very tedious and often inaccurate^23^. The n-alkane method is limited when the animal consumes a large number of plant species^24^. When the n-alkane method was used to determine diet composition of grazing yaks on the Tibetan plateau, only the two or three main dietary components could be detected^20^. Studies of DNA metabarcoding identified the level of forb dominance in megafaunal diets from permafrost sediment samples^25^ and allowed fine-grained niche separation from seven large mammalian herbivores^26^. These studies indicated that ingested plants can be effectively identified and quantified using the DNA metabarcoding method^27^.

This present study provides biological insights into the clusters and functionality of gut enterotypes. In particular, we describe gut enterotypes as well as the functional genomic information following seasonal dietary changes in free grazing yaks. The *Ruminococcaceae_UCG-005* enterotype was associated with high protein and low fiber diets in the warm season and the *Akkermansia* and uncultured *Eubacterium WCHB1-41* enterotype with low protein and high fiber diets in the cold season. It was suggested that the proportion of protein and carbohydrate contents in the diet mediates the host’s enterotype shift, at least in baboons^28,29^. Seasonal variations in dietary protein and carbohydrate contents could provide an attractive explanation in terms of enterotype dynamics, and also contribute to determine the enterotype for high altitude herbivores. Gut enterotype in humans remained stable when on a 10-day low-fat/high-fiber diet intervention^30^. However, the most compelling evidence for seasonal diet shifts in enterotypes in the present study was that enterotypes 1 and 2 shifted consistently between seasons and *Ruminococcaceae_UCG-01* enterotype 3 remained stable throughout the year. This might be the result of long-term co-evolution between the host and environment and suggests that the fixed seasonal enterotype dynamics play a key role in species formation and adaptation of mammals to the extreme environment at high altitude.

*Akkermansia* resides in the mucus layer of the gut and is known for its degradation of mucin^9,31^. It was reported that *A. muciniphila* plays a pivotal role in the prevention of obesity and type 1 and type 2 diabetes and, hence, promotes health in humans^32,33^. In addition, there is evidence that *A. muciniphila* functions as an energy sensor and regulates energy homeostasis for host microbial mutualism^34,35^. The present study demonstrated a substantial increase in *Akkermansia* and uncultured *Eubacterium WCHB1-41* enterotype 1 with low nitrogen and low energy intake in the cold season, suggesting that this enterotype responds to a high fiber and low protein diet. Previous studies reported that a high fat diet decreased mucus thickness^32^, whereas, a fiber-rich diet increased mucus thickness, which improved the gut positive barrier function^36^. *Akkermansia* and uncultured *Eubacterium WCHB1-41* degraded mucins and converted them into short-chain fatty acids (SCFAs), which provided nutrients for other resident bacteria and cells^37^.

Genetic studies in yaks demonstrated enrichments in the amino-acid metabolism gene (*Whsc1*, *Glul*), fatty acid biosynthesis and metabolism gene (*Hsd17b12*)^3^, and up-regulation in 36 genes that were related to volatile fatty acids transport and absorption in the ruminal epithelium of high-altitude ruminants^4^. These studies provide additional adaptive responses to insufficient energy intake that yaks experience. In addition, it was reported that yaks have a relatively low N requirement and utilize dietary N more efficiently than cattle in the cold season^38–41^. The current study also provides a mechanistic explanation as *Akkermansia* and Kiritimatiellaeota are involved in arginine and fatty acid biosynthesis pathways to utilize dietary N and generate energy in the cold season. In the context of the increased metabolic disease prevalence, this study provides an insight on the influence of *Akkermansia* and uncultured *Eubacterium WCHB1-41* enterotype in nitrogen and energy utilization in the host, and implies that this enterotype plays a key role in mediating the nutritional homeostasis in high altitude animals with large therapeutic potential of metabolic diseases in humans. Further analyses of genomic functions of gut microbiota of yaks would be beneficial for a better understanding of mammals living in an extreme environment. Findings in the present study demonstrate that gut microbiota respond to seasonal dietary shifts, which allows yaks to better utilize poor forage of low protein content. The understanding of diet–gut microbiota interaction improves our understanding of how yaks adapt to extreme environments.

## Methods

### Fecal sample collection and processing

The Tibetan plateau forms the high-altitude core of Asia and presents a challenge for mammals to survive^1^. In this study, yaks grazed on the Tibetan plateau in both TH and OCG regimes (Supplementary Fig. 1). In OCG, the yaks grazed freely on the same area at 3010–3300 m above sea level (2000 ha; 37°10′–37°12′N, 102°44′–102°51′E) year-round. Dominant and associated plant species included *Kobesia humilis*, *K. capillifolia*, *Polygonum viviparum*, *Stipa capillata*, *Elymus nutans*, *Thalictrum alpinum*, *Medicago ruthenica*, and *Artemisia smithii*. In the TH regime, yaks grazed pasture at 2930–3000 m above sea level (13.3 ha; 37°12′N, 102°46′E) in winter-spring; at 3130–3300 m above sea level (666.7 ha; 37°10′N, 102°44′E) in summer; and at 3015–3100 m above sea level (11.3 ha; 37°11′N, 102°44′E) in autumn. The dominant and associated species in winter-spring pasture included *E. nutans*, *K. humilis*, *K. capillifolia*, *S. capillata*, *Leymus secalinus*, *P. viviparum*, and *A. smithii*; in summer included *P. fruticose*, *P. viviparum*, *Juncus himalensis*, *Deschampsia cespitosa*, *Festuca ovina*, *Saussurea amara*, *Carex atrofusca*, and *C. moorcroftii*; in autumn included *P. fruticose*, *Salix oritrepha*, *P. viviparum*, *E. nutans*, *D. cespitosa*, *K. tibetica*, *K. humilis*, *C. kansuensis*, *J. castaneus*, *P. anserine*, *T. alpinum*, and *S. katochaete*. The annual mean temperature was −0.1 °C in the TH and 0 °C in the OCG with a peak in summer (June–August) and a trough in winter (December–February). The grassland was alpine meadow and alpine shrub meadow and the plant growing season was 90–120 days.

According to previous studies^42,43^, and the average monthly temperature and precipitation recoded by a nearby meteorological station in the study area, four seasons can be identified as spring (April–May), summer (June–August), autumn (September–October), and winter (November–March the following year) (Supplementary Fig. 2). In this study, sampling periods spanned the four seasons in 2017, namely, spring (May), summer (August), autumn (October), and winter (December) (for TH: *n* = 32, spring; *n* = 33, summer; *n* = 37, autumn; *n* = 45, winter and for OCG: *n* = 31, spring; *n* = 39, summer; *n* = 38, autumn; *n* = 47, winter). Fresh yak feces were collected from the TH and OCG grasslands, mixed thoroughly in an unused freezing tube, placed immediately into liquid nitrogen containers in the field, and transported to Lanzhou University until further processing. An amount of 0.2 g of fresh feces was used for DNA extraction with QIAamp^®^ Fast DNA Stool Mini Kit (50, QIAgen GmbH) and an extraction blank was processed to monitor for cross-contamination. DNA was quantified using the NanoDrop-2000 UV–Vis Spectrophotometer (Thermo Scientific, Wilmingtoo, DE, USA). The DNA samples were used for both diet (*n* = 302) and microbiota (*n* = 300) analyses. The studies and all procedures involving the animals were approved by experimental field management protocols (EAF2021012) of Lanzhou University.

### AGB and chemical composition analysis

Three plots (100 m × 100 m) were selected randomly in each of the TH and OCG grasslands in each of the four seasons. Within each plot, five quadrats (50 cm × 50 cm) were selected randomly. AGB was harvested in each quadrat, oven-dried at 65 °C to a constant weight, ground to pass through a 1-mm screen and stored at room temperature for chemical composition analysis. Dry matter (DM) was determined by oven-drying at 105 °C for 24 h, EE by extraction with petroleum ether and N content by the Kjeldahl method^44^. NDF and ADF were measured according to Van Soest et al.^45^, with heat stable alpha amylase and sodium sulfite used in the NDF procedure.

### Diet DNA metabarcoding

The P6 loop of the chloroplast *trn*L (UAA) region was used for DNA metabarcoding with primers *trn*L (UAA) g and *trn*L (UAA) h^16,25,26,46^ (Supplementary Table 1). For the PCR assays, 10 μL reactions of each of 0.3 μL primers, 0.2 μL KOD FX Neo, 2 μL dNTP, 5 μL KOD FX Neo buffer, and 50 ng of DNA template were mixed. Thermocycling followed a program of initial denaturing at 95 °C for 4 min, followed by 35 cycles of 94 °C for 30 s, 50 °C for 30 s, and 72 °C for 1 min, with a 5-min final extension at 72 °C. All PCRs were conducted with a no-template negative control and a positive control (consisting of DNA extracted from plant species from our local DNA reference library). The 5′ end of each primer was tagged by a 16-nt multiplex identification tag that differed by 8-nt from the other tag, allowing uniquely tagged PCR products. The sequence was carried out on Illumina HiSeq 2500 platform.

### 16S rRNA gene Illumina sequencing

The V3–V4 region of the 16S rRNA gene was sequenced on Illumina MiSeq 2500 platform with primers (341F/806R)^47^ (Supplementary Table 1). For the PCR assays, 50 μL of each of the 30 ng DNA template, fusion primer, and PCR master mix were mixed. The PCR cycles started with a 3 min denaturation at 94 °C, followed by 30 cycles each consisting of 94 °C for 30 s, 56 °C for 45 s, 72 °C for 45 s, and followed by a final step of 72 °C for 10 min. PCR products were purified with AmpureXP beads and eluted in elution buffer. Libraries were qualified by the Agilent 2100 bioanalyzer (Agilent, USA). The amplicons were sequenced on Illumina MiSeq 2500 and generated 2 × 300 bp paired-end reads.

### Reference plant DNA libraries

To identify diet plant sequences from fecal samples, we established an extensive DNA reference database from plant species throughout the alpine grassland area of the study, Yongfengtan and Wushaoling. The collection included 212 species that were most abundant in the study area. All plants were identified to species-level by expert botanists.

Reference plant DNA was extracted with DNeasy Plant Mini Kit (50, QIAgen GmbH) using 0.2 g of leaves, and sequenced chloroplast *trn*L-P6 (UAA) using established primers and protocols (Supplementary Table 1)^26,48^. DNA was quantified using the NanoDrop-2000 UV–Vis Spectrophotometer (Thermo Scientific, Wilmingtoo, DE, USA). *trn*L (UAA) was sequenced in 25 μL PCR reaction that included 2.5 μL MgCl_2_, 4 μL dNTP, 0.5 μL of each primer (*trn*L(UAA)c/*trn*L(UAA)d), 12.5 μL 2 × GC Buffer I, 0.25 μL *TaKaRa* LA Taq^®^, and 0.5 μL DNA template. Thermocycling for *trnL* (UAA) proceeded at 94 °C for 1 min, 35 cycles of 94 °C for 30 s, 56 °C for 30 s, and 72 °C for 1 min, with a 5 min extension at 72 °C. Programs Geneious and MEGA7.0.14 were used for sequence alignment and analysis.

### DNA metabarcoding sequence analysis

Sequence demultiplexing, quality, and preliminary identifications were conducted by QIIME 1.9.1. Demultiplexing used split_libraries_fastq.py^49^. Sequences shorter than 10 bp and mean Illumina fastq quality scores <20 were not considered. Paired reads were merged using USEARCH11^50^ and then all merged sequences of each sample were pooled. Quality filtering was performed on the pooled sequence with more than 0.5 expected error using fastq_filter command in USEARCH11 and only sequence lengths ≥ 10 were retained. Fastx_uniques command in USEARCH11 was used to find a set of unique sequences from filtered sequences, and singletons (sequence abundance = 1 across all samples) were removed. The remaining sequences were denoised (cluster at 100% similarity) using UNOISE algorithm^51^, during which OTU representative sequences were generated and potential chimeras were excluded. All the initially pooled sequences were mapped into the denoised sequence (zOTU) to generate an OTU table using otutab command implemented in USEARCH11. Strictly identical sequences were merged and assigned plant species based on their unique sequences to DNA metabarcode sequences with exact matches (100% identity) to reference sequences. Only unique sequences with an identity of 100% to reference sequences were kept for further analysis. When 100% identities were acquired from the local reference and NCBI libraries; preference was given to the local reference library. When a diet sequence matched multiple reference sequences exactly, assignments were revised to the finest taxonomic level by blasting with NCBI. We used the summarize_taxa command to group identical sequences, tally them within samples and then quantify the relative read abundance of each sequence, which is widely used to quantify the proportional foods consumed by animals^46,52–54^, and has been confirmed in several studies^26,55,56^. The resulting OUT counts per sample were rarefied to 40,000.

### Microbiota community analysis

Quality control, merging of pair ends, OTU clustering, and taxonomic assignation were performed using the QIIME 1.9.1. Illumina fastq quality scores < 20, ambiguous nucleotides and chimeras were discarded. The reads were assigned to OTUs using UNOISE3^51^ with a threshold of 100% identity and seeded with SILVA rRNA gene databases^57^. Sequences identified as archaea, mitochondria, and chloroplast were removed. After filtering and identification, the bacterial 16S rRNA gene data included sequences across 300 samples. The resulting OTU counts per sample were rarefied to 10,000.

### Application of enterotype clustering methodology

We applied methods described in humans^18,29,30,58^ to test for the presence of enterotypes in high-altitude yak. The genus-level relative abundance profiles of samples were clustered using Jensen–Shannon divergence (JSD) and BC dissimilarity and partitioning around medoid (PAM) clustering in R. The robustness of clusters was assessed by the Calinski–Harabasz (CH) index and silhouette score^59^. We applied the PAM, CH index, and silhouette score to clustering using BC and JSD methods, for which results did not differ (Supplementary Fig. 9). Furthermore, previous studies suggested that BC is related strongly to JSD^29,58^ and based on abundance method and suitable for revealing variations in abundance taxa, especially those with enterotypes. Thus, BC was implemented in genus-level abundance. To identify genus taxa contributing to enterotype groups based on BC, we applied the SIMPER method^29,60^, which identifies genus taxa contributing to similarity within- and dissimilarity between enterotypes and ranks their contribution.

### Statistical analysis

Standard R commands were performed to generate variations in relative abundance across seasons, and the Wilcoxon test (two-sample comparisons) or the Kruskal–Wallis test (multiple groups) was used to measure significance in non-parametric relative abundance profiles. The *t*-test was used to measure significance in AGB and chemical composition of the diet profiles compared with the average AGB or chemical composition across seasons. R was performed to visualize the seasonal dynamics in AGB and chemical composition of the diet as line chart and relative abundance of diet and microbiota taxa across seasons as streamplots or boxplots. We used BC dissimilarity and nonmetric multidimensional scaling (NMDS) in *vegan*^61^. Pairwise differences within and across seasonal diets and microbiota variation were permutested with 9999 permutations and false discovery rate (FDR) correction. To assess which plant and microbiota taxa were most responsible for seasonal differences in diet and microbiota variations, we performed indicator species analysis^62^ with *indicspecies* in R. We used the multipatt function with 9999 permutations to the list of species that were related with a group of samples and r.g. function determined the correlation between two binary vectors. Within seasons, the relationship between diet and microbiota richness in each sample was assessed by linear regression using the data across seasons in TH and OCG regimes. The correlations between diet and microbiota composition were computed based on Procrustes analysis, a program that compares the relative positions of points in two multivariate datasets^63^, and conducted in R using the *vegan* package. Monte Carlo *p*-values for rotational agreement significance testing were determined from 9999 permutations. For enterotype comparisons, samples were pooled into bins (spring, summer, autumn, and winter), and the significance among seasons were identified using Fisher’s exact test with FDR correction of *p*-values. FDR was applied at a level of 0.05 per tested correlation and significance for multiple comparison.

### Reporting summary

Further information on research design is available in the Nature Research Reporting Summary linked to this article.

## Supplementary information

Supplementary Information

Reporting Summary

## Data Availability

Illumina data are available at NCBI (BioProject ID: PRJNA650175).

## Code availability

Code and detailed information are available on github (https://github.com/wuqunfu/Seasonal-dynamics-of-gut-microbiota-of-yaks.git).

## References

[1] Meyer MC (2017). Permanent human occupation of the central Tibetan Plateau in the early Holocene. Science.

[2] Qiu Q (2015). Yak whole-genome resequencing reveals domestication signatures and prehistoric population expansions. Nat. Commun..

[3] Qiu Q (2012). The yak genome and adaptation to life at high altitude. Nat. Genet..

[4] Zhang Z (2016). Convergent evolution of rumen microbiomes in high-altitude mammals. Curr. Biol..

[5] Johnson AJ (2019). Daily sampling reveals personalized diet–microbiome associations in humans. Cell Host Microbe.

[6] Ren T (2017). Seasonal, spatial, and maternal effects on gut microbiome in wild red squirrels. Microbiome.

[7] Sonnenburg JL, Backhed F (2016). Diet–microbiota interactions as moderators of human metabolism. Nature.

[8] Heijtz RD (2011). Normal gut microbiota modulates brain development and behavior. Proc. Natl Acad. Sci. USA.

[9] Smits SA (2017). Seasonal cycling in the gut microbiome of the Hadza hunter-gatherers of Tanzania. Science.

[10] Maurice CF (2015). Marked seasonal variation in the wild mouse gut microbiota. ISME J..

[11] Xue ZS (2015). The bamboo-eating giant panda harbors a carnivore-like gut microbiota, with excessive seasonal variations. mBio.

[12] Hicks AL (2018). Gut microbiomes of wild great apes fluctuate seasonally in response to diet. Nat. Commun..

[13] Gomez A (2016). Temporal variation selects for diet-microbe co-metabolic traits in the gut of *Gorilla* spp. ISME J..

[14] Bergmann GT, Craine JM, Robeson MS, Fierer N (2015). Seasonal shifts in diet and gut microbiota of the American bison (*Bison bison*). PLoS ONE.

[15] Kartzinel TR, Hsing JC, Musili PM, Brown BRP, Pringle RM (2019). Covariation of diet and gut microbiome in African megafauna. Proc. Natl Acad. Sci. USA.

[16] Taberlet P (2007). Power and limitations of the chloroplast *trn*L (UAA) intron for plant DNA barcoding. Nucleic Acids Res..

[17] Feng, H. Y. & Pan, J. B. *Field Guide to Wild Plants of China (Qilian Mountains)* (The Commercial Press, 2015).

[18] Costea PI (2018). Enterotypes in the landscape of gut microbial community composition. Nat. Microbiol..

[19] Guo W (2020). Survey of rumen microbiota of domestic grazing yak during different growth stages revealed novel maturation patterns of four key microbial groups and their dynamic interactions. Anim. Microbiome.

[20] Ding LM, Long RJ (2010). The use of herbage n-alkanes as markers to estimate the diet composition of yaks on the Qinghai-Tibetan Plateau.. Asian-Australasian J. Anim. Sci..

[21] Newmaster SG (2013). Examination of two new technologies to assess the diet of woodland caribou: video recorders attached to collars and DNA barcoding. Can. J. For. Res..

[22] Kleynhans EJ, Jolles AE, Bos MRE, Olff H (2011). Resource partitioning along multiple niche dimensions in differently sized African savanna grazers. Oikos.

[23] Carriere S (2002). Photographic Key for the microhistological identification of some Arctic vascular plants. Arctic.

[24] Dove H, Mayes RW (2006). Protocol for the analysis of n-alkanes and other plant-wax compounds and for their use as markers for quantifying the nutrient supply of large mammalian herbivores. Nat. Protoc..

[25] Willerslev E (2014). Fifty thousand years of Arctic vegetation and megafaunal diet. Nature.

[26] Kartzinel TR (2015). DNA metabarcoding illuminates dietary niche partitioning by African large herbivores. Proc. Natl Acad. Sci. USA.

[27] Poinar HN (1998). Molecular coproscopy: dung and diet of the extinct ground sloth *Nothrotheriops shastensis*. Science.

[28] Ren T, Grieneisen LE, Alberts SC, Archie EA, Wu M (2016). Development, diet and dynamism: longitudinal and cross-sectional predictors of gut microbial communities in wild baboons. Environ. Microbiol..

[29] Wang J (2014). Dietary history contributes to enterotype-like clustering and functional metagenomic content in the intestinal microbiome of wild mice. Proc. Natl Acad. Sci. USA.

[30] Wu GD (2011). Linking long-term dietary patterns with gut microbial enterotypes. Science.

[31] Sonnenburg ED, Sonnenburg JL (2019). The ancestral and industrialized gut microbiota and implications for human health. Nat. Rev. Microbiol..

[32] Everard A (2013). Cross-talk between *Akkermansia muciniphila* and intestinal epithelium controls diet-induced obesity. Proc. Natl Acad. Sci. USA.

[33] Hansen CH (2012). Early life treatment with vancomycin propagates *Akkermansia muciniphila* and reduces diabetes incidence in the NOD mouse. Diabetologia.

[34] Chevalier C (2015). Gut microbiota orchestrates energy homeostasis during cold. Cell.

[35] Preidis GA (2015). Composition and function of the undernourished neonatal mouse intestinal microbiome. J. Nutr. Biochem..

[36] Desai MS (2016). A dietary fiber-deprived gut microbiota degrades the colonic mucus barrier and enhances pathogen susceptibility. Cell.

[37] Derrien M, Vaughan EE, Plugge CM, de Vos WMD (2004). *Akkermansia muciniphila* gen. nov., sp. nov., a human intestinal mucin-degrading bacterium. Int. J. Syst. Evol. Microbiol..

[38] Zhou JW (2017). Comparison of nitrogen utilization and urea kinetics between yaks (*Bos grunniens*) and indigenous cattle (*Bos taurus*). J. Anim. Sci..

[39] Wang HC (2011). Comparison of nitrogen metabolism in yak (*Bos grunniens*) and indigenous cattle (*Bos taurus*) on the Qinghai-Tibetan Plateau. Asian-Australasian J. Anim. Sci..

[40] Guo XS (2012). Nitrogen metabolism and recycling in yaks (*Bos grunniens*) offered a forage-concentrate diet differing in N concentration. Anim. Prod. Sci..

[41] Long RJ (2004). Digestibility, nutrient balance and urinary purine derivative excretion in dry yak cows fed oat hay at different levels of intake. Livest. Prod. Sci..

[42] Xu C, Ma YM, You C, Zhu ZK (2015). The regional distribution characteristics of aerosol optical depth over the Tibetan Plateau. Atmos. Chem. Phys..

[43] Tang MC, Reiter ER (1984). Plateau monsoons of the northern hemisphere: a comparison between North America and Tibet. Mon. Weather Rev..

[44] Horwitz W, Latimer GW (2010). Official Methods of Analysis of AOAC International.

[45] Van Soest PV, Robertson J, Lewis BA (1991). Methods for dietary fiber, neutral detergent fiber, and nonstarch polysaccharides in relation to animal nutrition. J. Dairy Sci..

[46] De Barba M (2014). DNA metabarcoding multiplexing and validation of data accuracy for diet assessment: application to omnivorous diet. Mol. Ecol. Resour..

[47] Takahashi S (2014). Development of a prokaryotic universal primer for simultaneous analysis of Bacteria and Archaea using next-generation sequencing. PLoS ONE.

[48] Taberlet P, Gielly L, Pautou G, Bouvet J (1991). Universal primers for amplification of three non-coding regions of chloroplast DNA. Plant Mol. Biol..

[49] Kuczynski J (2011). Using QIIME to analyze 16S rRNA gene sequences from microbial communities. Curr. Protoc. Bioinforma..

[50] Edgar RC (2013). UPARSE: highly accurate OTU sequences from microbial amplicon reads. Nat. Methods.

[51] Edgar, R. C. UNOISE2: improved error-correction for Illumina 16S and ITS amplicon sequencing. Preprint at *bioRxiv*, 10.1101/081257 (2016).

[52] Cachera M, Ernande B, Villanueva MC, Lefebvre S (2017). Individual diet variation in a marine fish assemblage: optimal foraging theory, niche variation hypothesis and functional identity. J. Sea Res..

[53] Lopes CM (2015). DNA metabarcoding diet analysis for species with parapatric vs sympatric distribution: a case study on subterranean rodents. Heredity.

[54] Craine JM, Towne EG, Miller M, Fierer N (2015). Climatic warming and the future of bison as grazers. Sci. Rep..

[55] Pansu J (2019). Trophic ecology of large herbivores in a reassembling African ecosystem. J. Ecol..

[56] Srivathsan A, Sha JC, Vogler AP, Meier R (2015). Comparing the effectiveness of metagenomics and metabarcoding for diet analysis of a leaf-feeding monkey (*Pygathrix nemaeus*). Mol. Ecol. Resour..

[57] Quast C (2013). The SILVA ribosomal RNA gene database project: improved data processing and web-based tools. Nucleic Acids Res..

[58] Arumugam M (2011). Enterotypes of the human gut microbiome. Nature.

[59] Hildebrand F (2013). Inflammation-associated enterotypes, host genotype, cage and inter-individual effects drive gut microbiota variation in common laboratory mice. Genome Biol..

[60] Clarke KR (1993). Non-parametric multivariate analyses of changes in community structure. Aust. J. Ecol..

[61] Oksanen, J. et al. Vegan: community ecology package. *R Package Version 1.17-4* http://CRAN.R-project.org/package=vegan (2010).

[62] Caceres MD, Legendre P (2009). Associations between species and groups of sites: indices and statistical inference. Ecology.

[63] Muegge BD (2011). Diet drives convergence in gut microbiome functions across mammalian phylogeny and within humans. Science.

