# Supplementary material for: Seasonal dynamics of diet–gut microbiota interaction in adaptation of yaks to life at high altitude
Source: NPJ Biofilms Microbiomes. 2021 Apr 20;7:38. doi: 10.1038/s41522-021-00207-6 (PMC8058075; doi:10.1038/s41522-021-00207-6)
Supplement: Supplementary file 1 — Supplementary Information [file 41522_2021_207_MOESM1_ESM.pdf]

Supplementary Figure 1. The location of the sampling sites on the northeastern Tibetan Plateau. The vegetation map was acquired from China's vegetation atlas with a scale of 1:1,000,000 (Inserted image)[1]. The sampling sites was obtained from Google earth.

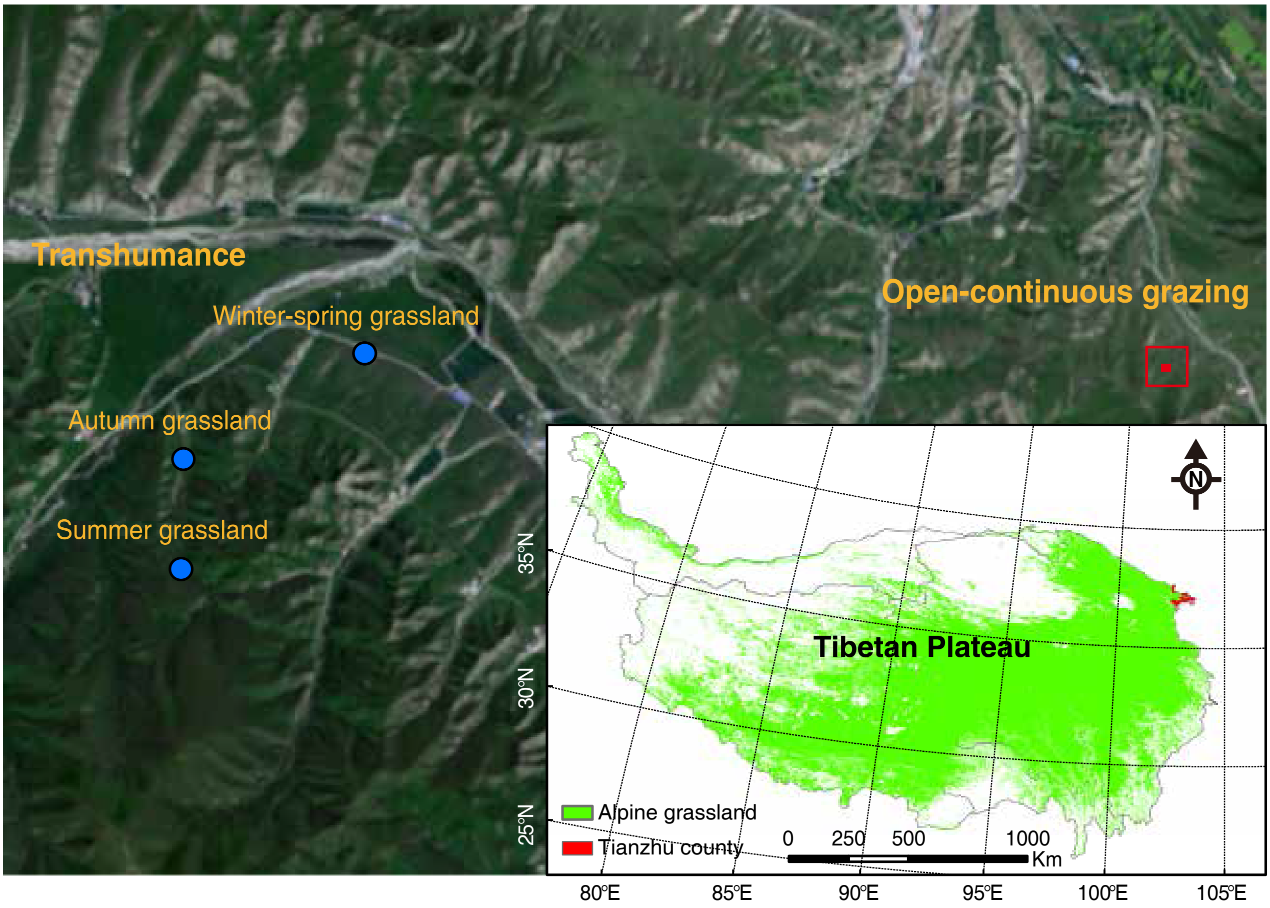

Supplementary Figure 2. Four seasons distinguished by average monthly precipitation and temperature for sampling region in 2017. The data were provided by the National Meteorological Administration of China.

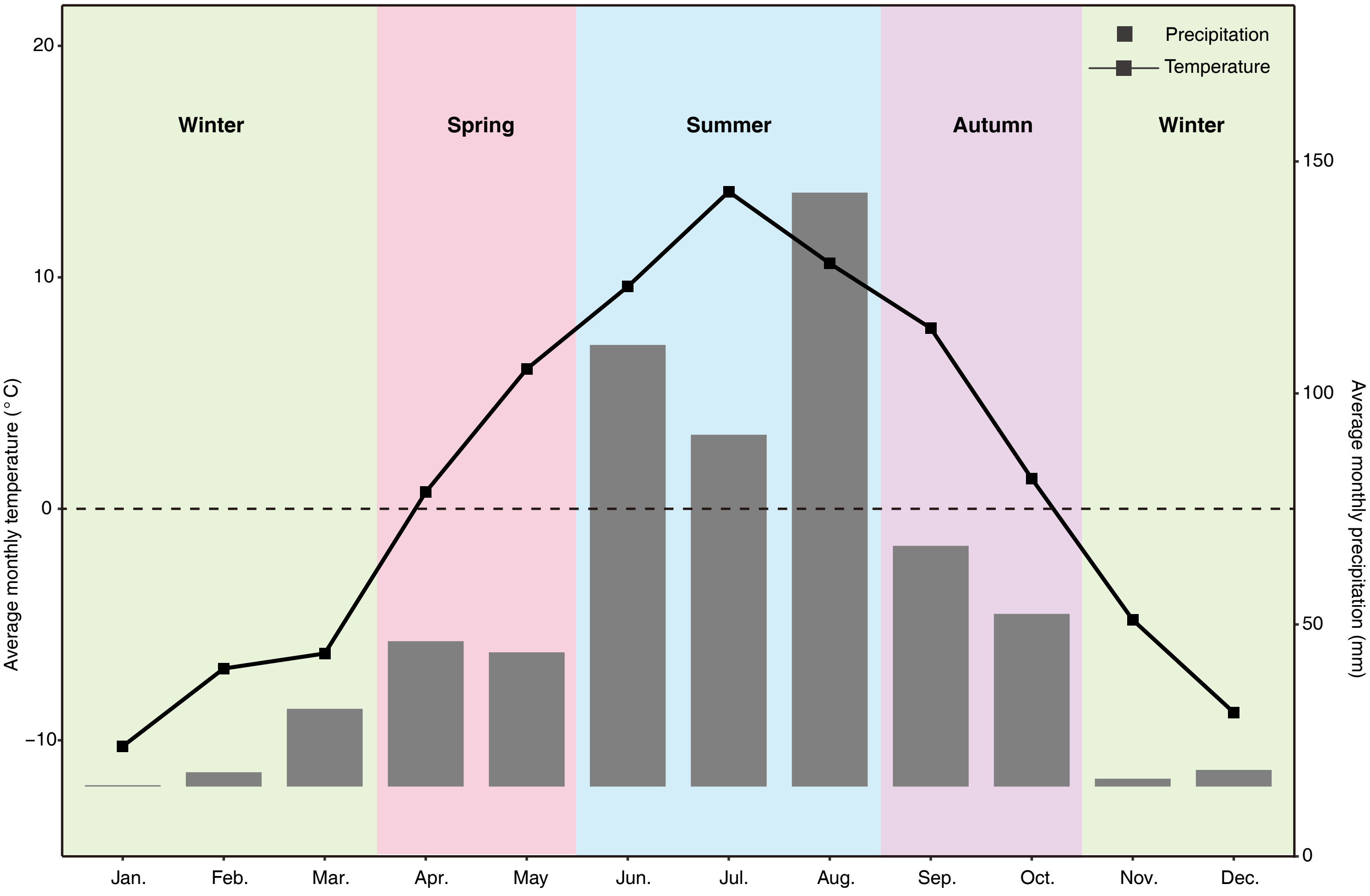

Supplementary Figure 3. Seasonal dynamics in above-ground biomass (AGB) and chemical composition (dry matter basis) of the diet in transhumance grassland. (**a**) Line chart represents the AGB (kg DM/ha) in the four seasons. The dashed line is mean AGB in the four seasons. (**b**) Line chart represents chemical composition (crude protein (CP), ether extract (EE), acid detergent fiber (NDF) and neutral detergent fiber (ADF)) of diets in the four seasons. The dashed lines are the mean of each chemical composition in the four seasons and are colored by each chemical composition. Values and error bars are shown as mean ± SE. Average contents of CP are negatively correlated with those of NDF (R^2=0.23, p<0.05) (**c**) and ADF (R^2=0.79, p<0.001) (**d**). Statistic tests are performed by the t-test with FDR (false discovery rate) corrected p-value. p-values, * < 0.05, ** < 0.01, *** < 0.001.

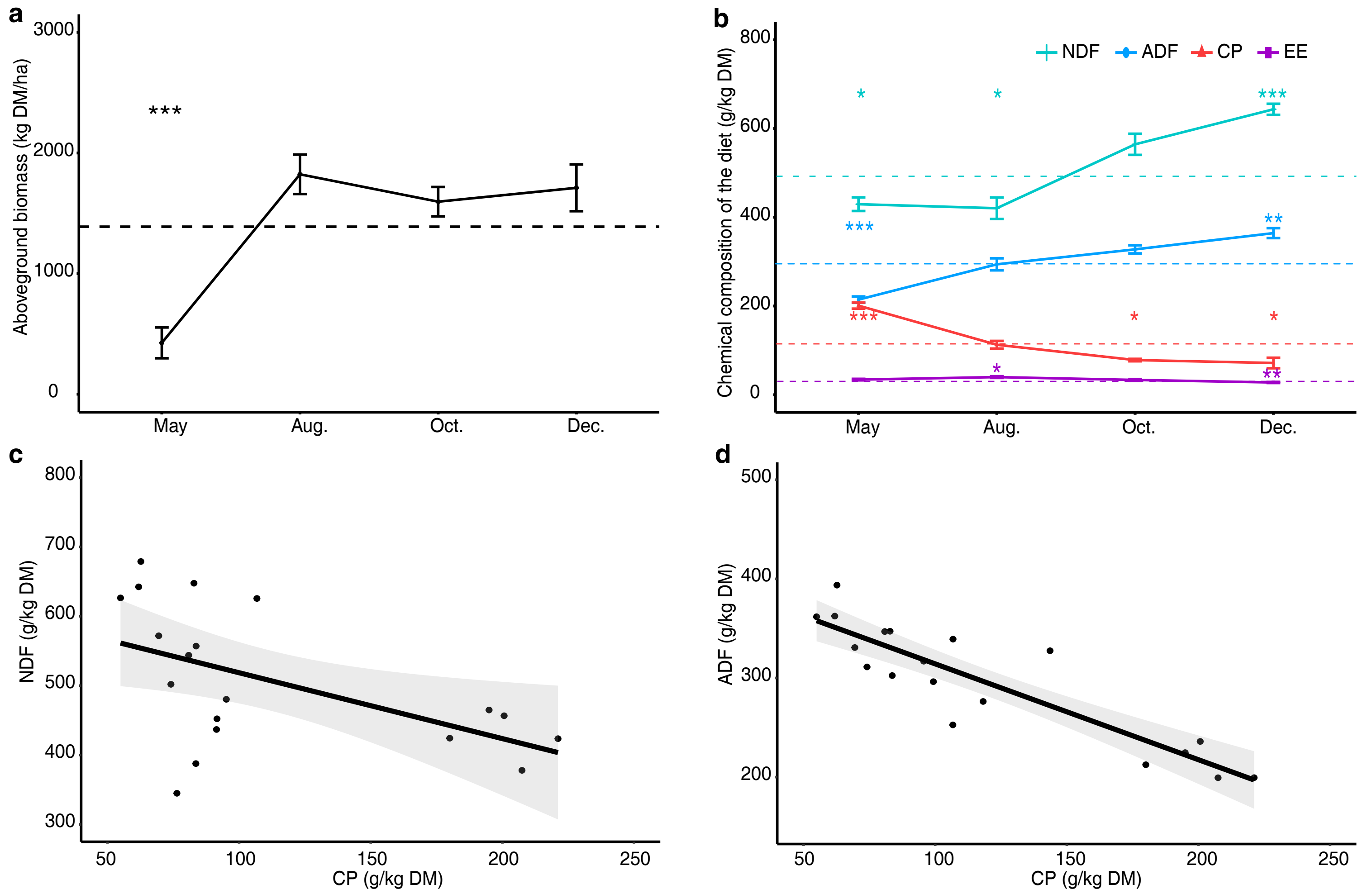

Supplementary Figure 4. Nonmetric multidimensional scaling (NMDS) based on the Bray-Curtis dietary and gut microbiota dissimilarity. Within and among seasons Bray-Curtis dissimilarity in diet and microbiota are presented in Supplementary Table 2. The same ordinations display seasonal diet (left) and gut microbiota (right) compositions regardless of grazing regimes. Ordinations are based on (**a**) 302 dietary samples and (**b**) 300 microbiota samples. Difference in diet (stress=0.19, adonis pseudo R^2=0.75, p<0.001; anosim pseudo R=0.89, p<0.001) and microbiota (stress=0.16, adonis pseudo R^2=0.17, p<0.001; anosim pseudo R=0.45, p<0.001) compositions strongly reflect seasonal fluctuations. Analysis of similarities (ANOSIM) and permutational multivariate analysis of variance (PERMANOVA) were used for statistical testing of treatment similarities. Warm and cool colors distinguish transhumance (TH) and open-continuous grazing (OCG) regimes, respectively. The dotted ellipse borders represent the 95% confidence interval.

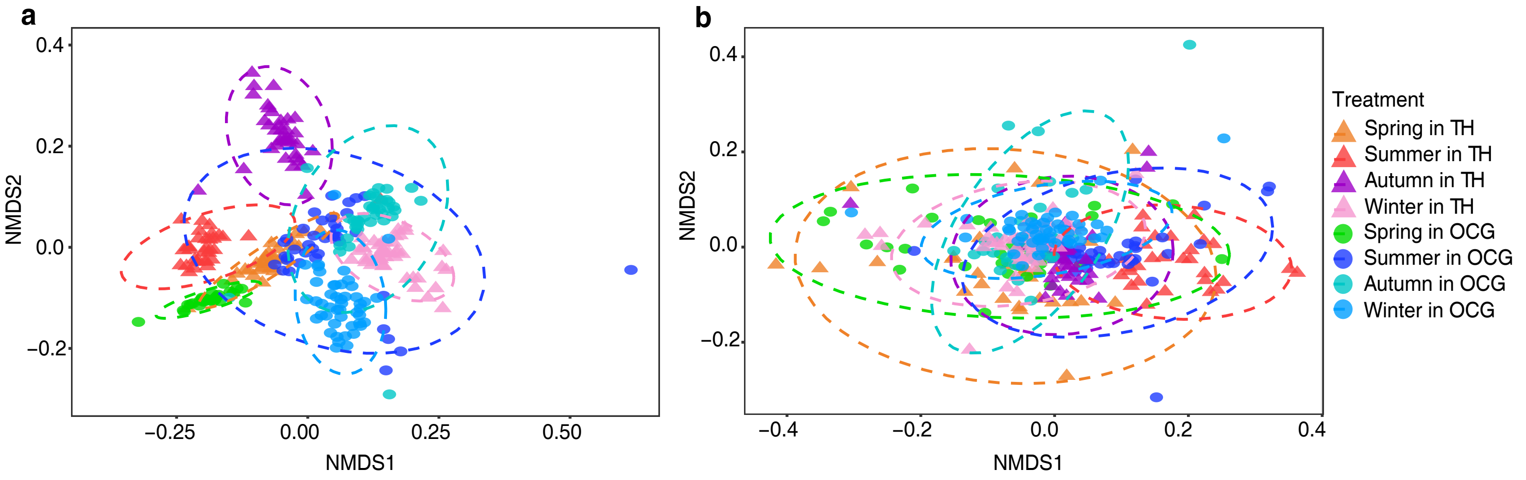

Supplementary Figure 5. Alpha-diversity metrics of yak diet and gut microbiota by season. Columns show the richness (left), Shannon diversity (middle), and Chao1 index (right). Diet (**a-f**) and gut microbiota (**g-l**) diversities are plotted by their distributions in transhumance (**a-c, g-i**) and open-continuous grazing (**d-f, j-l**) regimes. All boxplot distributions are tested by non-parametric Kruskal-Wallis and Wilcoxon with FDR (false discovery rate) corrected *p*-value, center values indicate the median and error bars. *p*-values, * < 0.05, *** < 0.01, *** < 0.001.

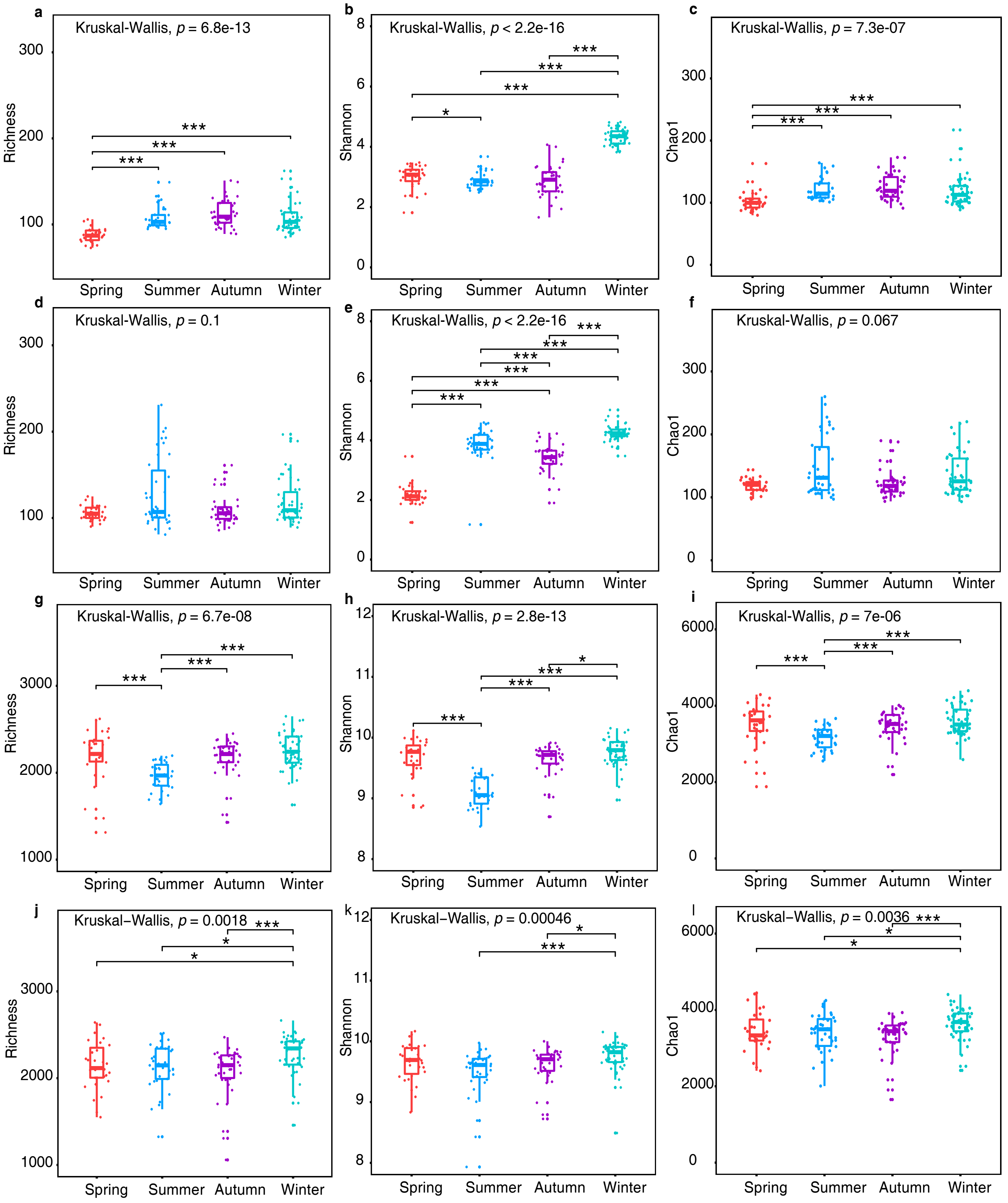

Supplementary Figure 6. Yak gut microbiota by season at the phylum level. Relative abundance of most phyla (>5%) over seasons (spring, summer, autumn and winter) are aggregated and colored on a stream-graph in (**a**) transhumance and (**c**) open-continuous grazing regimes. Indicator phyla that are related to each season are tracked using Sankey plots in (**b**) transhumance and (**d**) open-continuous grazing. Lines connect season to phylum level, which are colored by phylum level. Line widths are scaled to reflect indicator value (higher phylum indicator value is associated more strongly with the season). The statistical *p* values refer to the phylum associated with the season. *p*-values, * < 0.05, ** < 0.01, *** < 0.001.

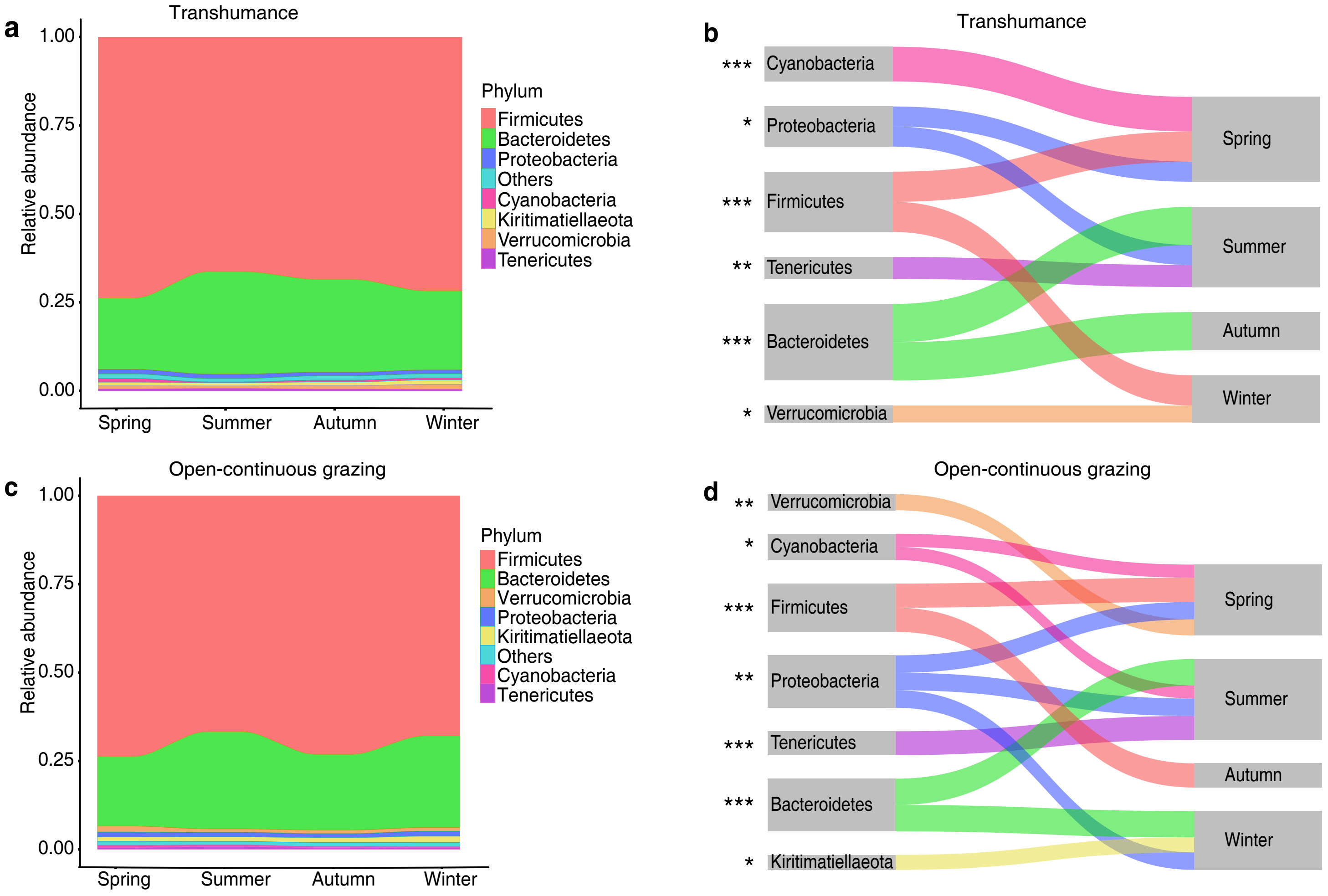

Supplementary Figure 7. Lollipop charts showing indicator species for the yak diet and gut microbiota. Columns denote the indicator species for transhumance (left) and open-continuous grazing (right) regimes. Each row organizes the data at family level of diet (**a-b**), phylum (**c-d**) and genus level (**e-f**) of gut microbiota. Lollipops are colored by different diet family or microbiota at phylum and genus level. A high value indicates a species has a high indicator power for the corresponding group. *p*-values, * < 0.05, ** < 0.01, *** < 0.001.

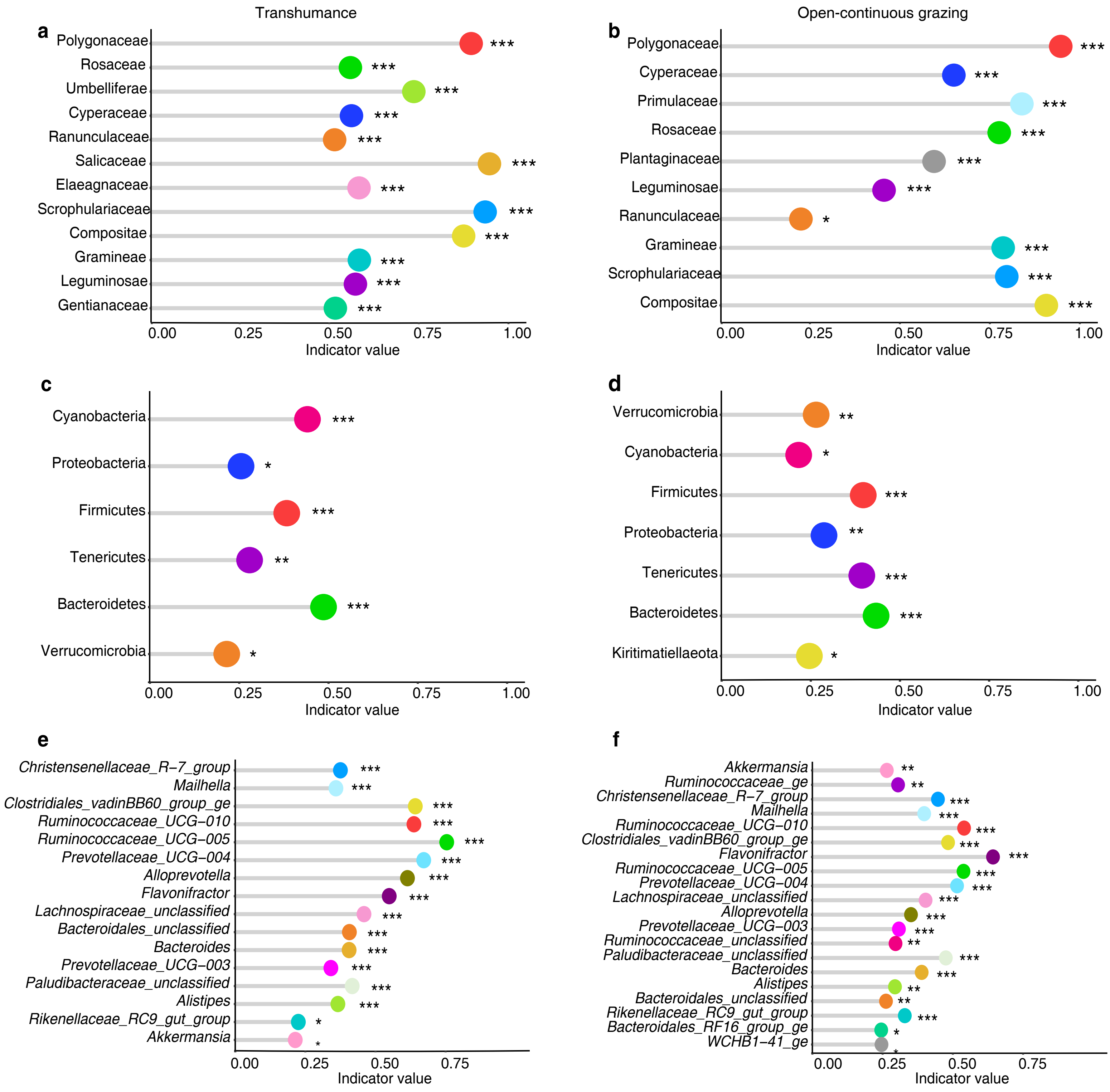

Supplementary Figure 8. Within-group correlations between diet and gut microbiota richness across seasons. There is no relationship between diet and gut microbiota richness within samples in each season and each grazing regime. TH and OCG represent transhumance and open-continuous grazing regimes, respectively.

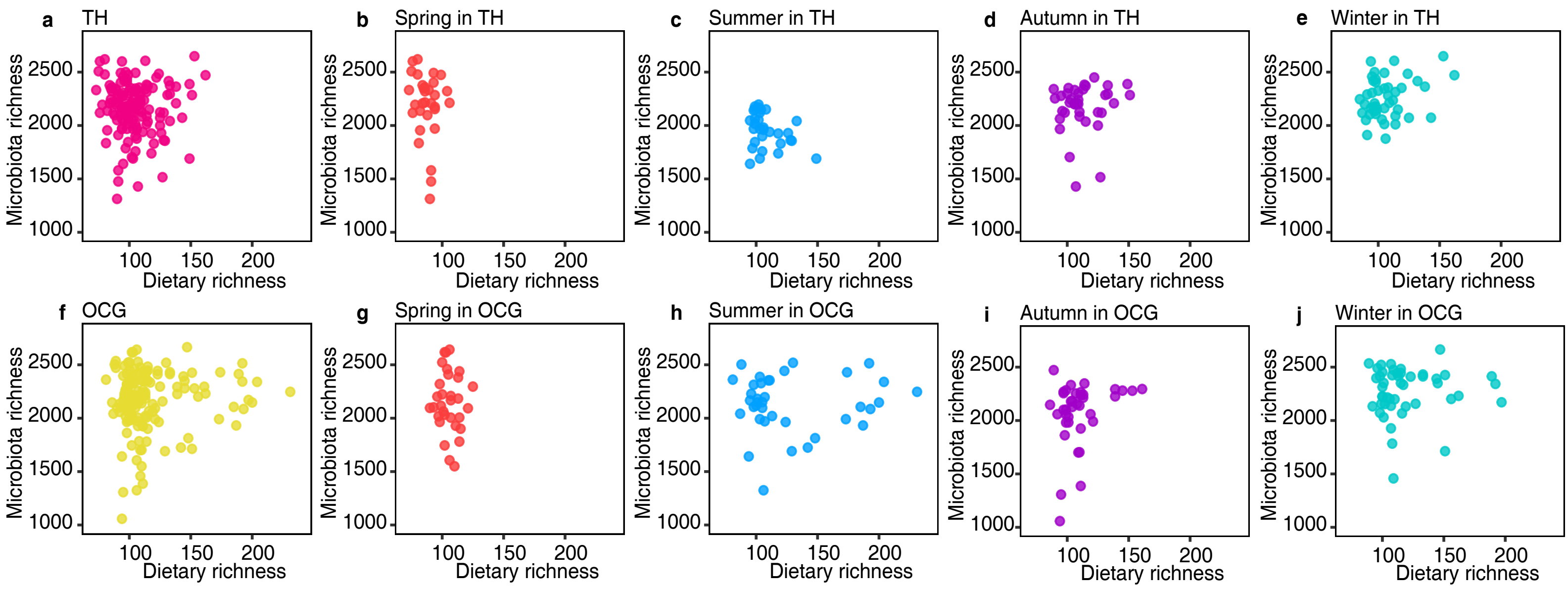

Supplementary Figure 9. Enterotype clusters identified by partitioning around medoid (PAM) based on the Bray-Curtis dissimilarity (BC dissimilarity) and Jensen-Shannon distance (JSD). Clustering of gut microbiota taxa into enterotypes are presented in main text Fig. 7. Principal coordinated analysis plots represent the three enterotype clusters of yak gut microbiota identified by PAM based on the (**a**) BC and (**f**) JSD among relative abundance at the genus level (297 samples). Numbered white rectangles denote the centroid of each cluster and solid lines denote the distance of each sample from the centroid of enterotype cluster. Boxplots represent of similarity (ANOSIM) analyses within and between enterotypes identified from (**b**) BC and (**g**) JSD based on relative abundance of genus level. Area charts represent the relative abundance of the top10 contributors based on the (**c**) BC and (**h**) JSD from different enterotypes. Boxplots represent the distribution of Shannon diversity index based on (**d**) BC and JSD (**i**) across different enterotypes. Optimal number of clusters for PAM analysis based on (**e**) BC and (**j**) JSD among the relative abundance distributions at the genus level. Columns denote the three enterotype clusters based on BC (left) and JSD (right). All boxplot distributions were tested by non-parametric Kruskal-Wallis and Wilcoxon with FDR (false discovery rate) corrected *p* value. Boxplot center values represent the median and error bars. Analysis of similarities (ANOSIM) and permutational multivariate analysis of variance (PERMANOVA) were used for statistical testing within and between enterotype similarities. The color key by the side of panel **h** applies to panel **c**. *p*-values, * < 0.05, ** < 0.01, *** < 0.001.

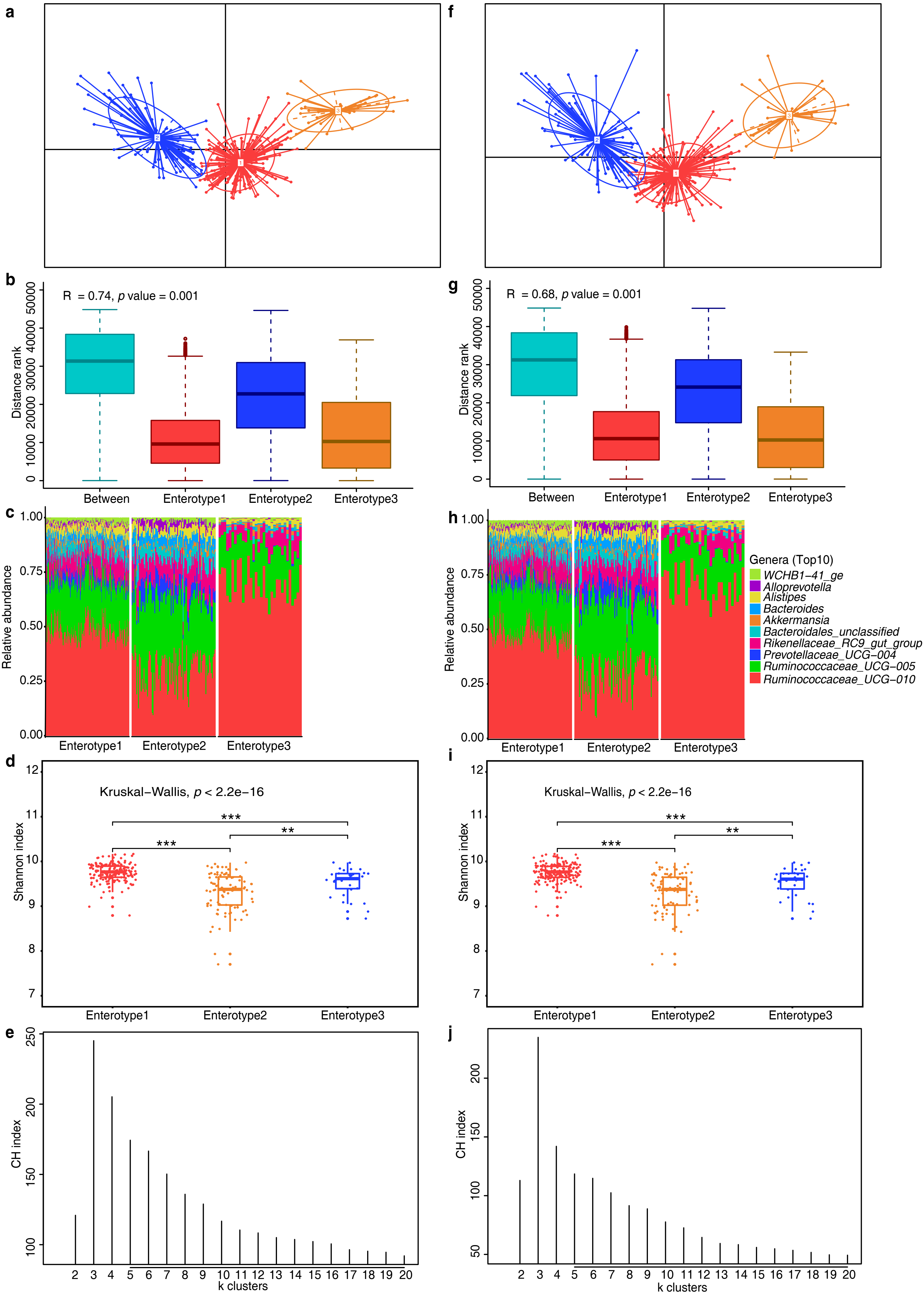

Supplementary Figure 10. The relative abundances corresponding to the main contributors at the gut microbiota genus level taxa. Boxplots represent microbiota genera that were sampled in spring, summer, autumn and winter. Boxplot center values represent the median and error bars. These ten genera were chosen based on their average contribution to overall Bray-Curtis dissimilarity. All boxplot distributions were tested by non-parametric Kruskal-Wallis and Wilcoxon with FDR (false discovery rate) corrected *p* value and center values indicate the median and error bars. Similar plots for *Ruminococcaceae_UCG_010*, *Ruminococcaceae_UCG_005*, *Akkermansia* and *WCH_I-41_ge* are presented in the main text Fig. 7. Enterotype clusters are presented in Supplementary Figure 9. *p*-values, * < 0.05, ** < 0.01, *** < 0.001.

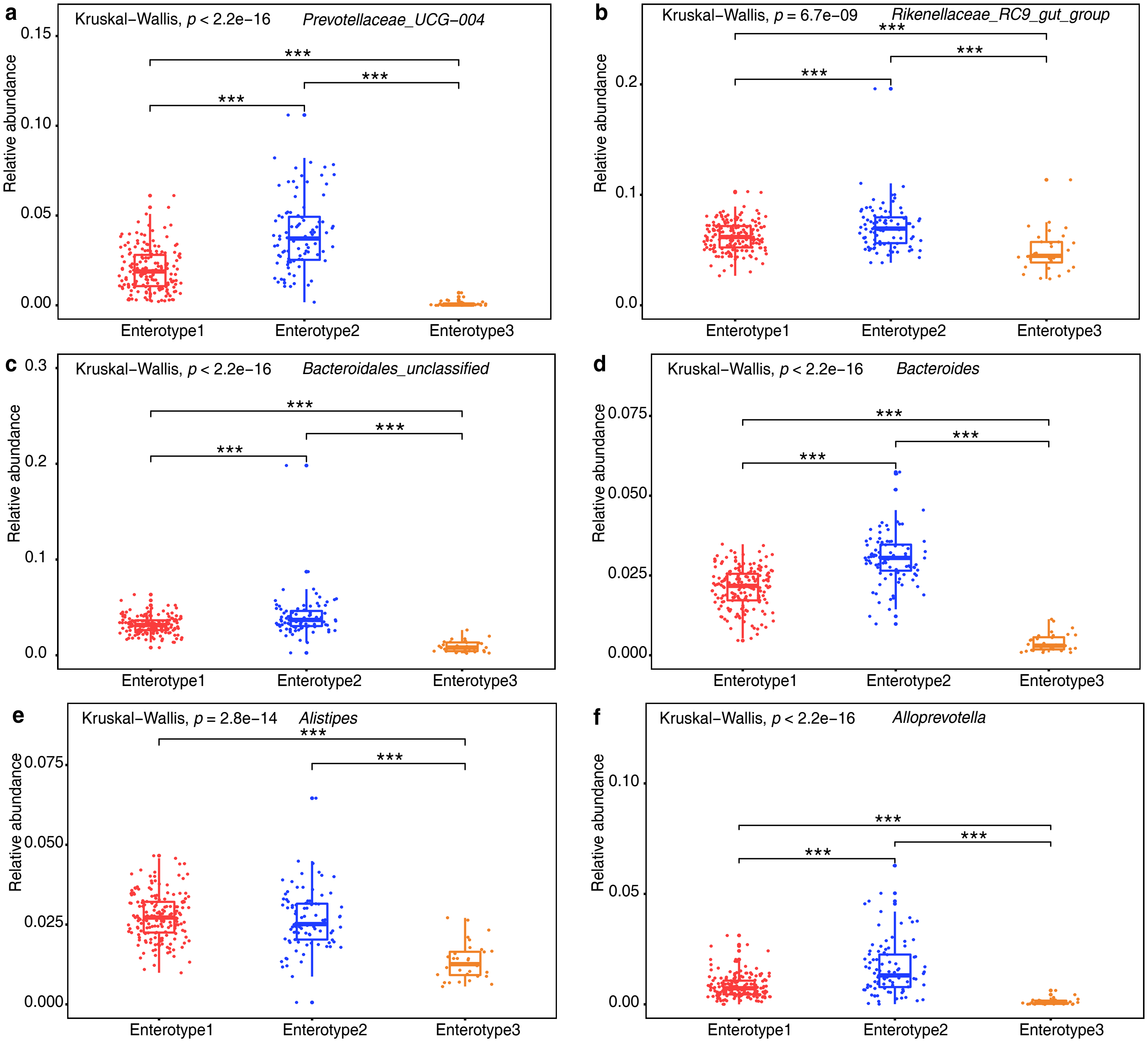

Supplementary Table 1. Primers utilized in this study.

Primer	Sequence	Reference
*trn*L (UAA) c	CGAAATCGGTAGACGCTACG	(48)
*trn*L (UAA) d	GGGGATAGAGGGACTTGAAC	(48)
*trn*L (UAA) g	GGGCAATCCTGAGCCAA	(16)
*trn*L (UAA) h	CCATTGAGTCTCTGCACCTATC	(16)
341F	ACTCCTACGGGAGGCAGCAG	(47)
806R	GGACTACHVGGGTWTCTAAT	(47)

Supplementary Table 2. Within and among seasons Bray-Curtis dissimilarity in diet and microbiota in transhumance and open-continuous grazing regimes. Pairwise distance matrices denote the mean weighted Bray-Curtis dissimilarity of diets (lower triangle) and microbiota (upper triangle) across all samples of each season. Values are weighted means of Bray-Curtis dissimilarities (Permutation test: $p < 0.001$). See plots corresponding to these data list in main text Fig. 1 and Supplementary Figure 4.

Grazing regimes	Season	Spring	Summer	Autumn	Winter	Within-seasons
Transhumance	Spring		0.545	0.489	0.481	0.486
	Summer	0.482		0.496	0.530	0.433
	Autumn	0.729	0.652		0.454	0.436
	Winter	0.544	0.779	0.714		0.436
	Within-seasons	0.225	0.240	0.216	0.313	
Open-continuous grazing	Spring		0.508	0.475	0.483	0.475
	Summer	0.682		0.473	0.483	0.434
	Autumn	0.734	0.575		0.459	0.436
	Winter	0.675	0.582	0.566		0.432
	Within-seasons	0.117	0.385	0.315	0.317	

Supplementary Table 3. Sample sizes and numbers of sequences after key steps of the data filtering protocol.

	Diet	Microbiota
Marker	*trn*L-P6	V3-V4
Fecal samples analyzed	302	300
High quality sequences	30534414	25375771
Unique sequences	227721	11895338
Unique sequences after removal of singleton	81530	1125235

Supplementary References

1. Chinese Academy of Sciences, 2001. Vegetation Atlas of China. Science Press, Beijing.
