# Supplementary material for: Seasonal dynamics of diet–gut microbiota interaction in adaptation of yaks to life at high altitude
Source: NPJ Biofilms Microbiomes. 2021 Apr 20;7:38. doi: 10.1038/s41522-021-00207-6 (PMC8058075; doi:10.1038/s41522-021-00207-6)
Supplement: Supplementary file 2 — Reporting Summary [file 41522_2021_207_MOESM2_ESM.pdf]

nature research

Corresponding author(s): NPJBIOFILMS-00933R Ruijun Long

Last updated by author(s): Mar 11, 2021

Reporting Summary

Nature Research wishes to improve the reproducibility of the work that we publish. This form provides structure for consistency and transparency in reporting. For further information on Nature Research policies, see our Editorial Policies and the Editorial Policy Checklist.

Statistics

For all statistical analyses, confirm that the following items are present in the figure legend, table legend, main text, or Methods section.

n/a	Confirmed	
☐	☒	The exact sample size (*n*) for each experimental group/condition, given as a discrete number and unit of measurement
☐	☒	A statement on whether measurements were taken from distinct samples or whether the same sample was measured repeatedly
☐	☒	The statistical test(s) used AND whether they are one- or two-sided<br>*Only common tests should be described solely by name; describe more complex techniques in the Methods section.*
☐	☒	A description of all covariates tested
☐	☒	A description of any assumptions or corrections, such as tests of normality and adjustment for multiple comparisons
☐	☒	A full description of the statistical parameters including central tendency (e.g. means) or other basic estimates (e.g. regression coefficient) AND variation (e.g. standard deviation) or associated estimates of uncertainty (e.g. confidence intervals)
☐	☒	For null hypothesis testing, the test statistic (e.g. *F*, *t*, *r*) with confidence intervals, effect sizes, degrees of freedom and *P* value noted<br>*Give P values as exact values whenever suitable.*
☐	☒	For Bayesian analysis, information on the choice of priors and Markov chain Monte Carlo settings
☐	☒	For hierarchical and complex designs, identification of the appropriate level for tests and full reporting of outcomes
☐	☒	Estimates of effect sizes (e.g. Cohen's *d*, Pearson's *r*), indicating how they were calculated

Our web collection on statistics for biologists contains articles on many of the points above.

Software and code

Policy information about availability of computer code

Data collection	The data collection was performed by Geneious, MEGA7.0.14, QIIME1.9.1 and R3.4.3 software. All software are open source. Code and detailed information are available on github (https://github.com/wuqunfu/Seasonal-dynamics-of-gut-microbiota-of-yaks.git).
Data analysis	The data analyses were performed by Geneious, MEGA7.0.14, QIIME 1.9.1 and R3.4.3 software. All software are open source. Code and detailed information are available on github (https://github.com/wuqunfu/Seasonal-dynamics-of-gut-microbiota-of-yaks.git).

For manuscripts utilizing custom algorithms or software that are central to the research but not yet described in published literature, software must be made available to editors and reviewers. We strongly encourage code deposition in a community repository (e.g. GitHub). See the Nature Research guidelines for submitting code & software for further information.

Data

Policy information about availability of data

All manuscripts must include a data availability statement. This statement should provide the following information, where applicable:

- Accession codes, unique identifiers, or web links for publicly available datasets
- A list of figures that have associated raw data
- A description of any restrictions on data availability

The Illumina data are available at NCBI(BioProject ID:PRJNA650175). The list of figures that have associated raw data were upload with raw data. There was no restrictions on the data availability.

Field-specific reporting

Please select the one below that is the best fit for your research. If you are not sure, read the appropriate sections before making your selection.

☐ Life sciences ☐ Behavioural & social sciences ☒ Ecological, evolutionary & environmental sciences

For a reference copy of the document with all sections, see nature.com/documents/nr-reporting-summary-flat.pdf

Ecological, evolutionary & environmental sciences study design

All studies must disclose on these points even when the disclosure is negative.

Study description	Sequencing plant trnL (UAA) region and 16S rRNA gene analysis were employed to determine dietary composition and gut microbiota in freely grazing yaks on the Tibetan Plateau.
Research sample	Yaks freely grazed on the Tibetan plateau in both transhumance (TH) and open-continuous grazing (OCG) regimes, and fresh feces were collected from yak (n=302).
Sampling strategy	Fresh yak feces were collected from the TH and OCG grasslands, mixed thoroughly in an unused freezing tube, placed immediately into liquid nitrogen containers in the field, and transported to Lanzhou University until further processing. According to the previous studies, the sample size is better more than 27 per species.
Data collection	Fresh yak feces were collected from the transhumance (TH) and open-continuous grazing (OCG) grasslands, mixed thoroughly in an unused freezing tube, placed immediately into liquid nitrogen containers in the field, and transported to Lanzhou University until further processing. An amount of 0.2 g of fresh feces was used for DNA extraction with QIAamp® Fast DNA Stool Mini Kit (50, QIAgen GmbH) and an extraction blank was processed to monitor for cross-contamination. DNA was quantified using the NanoDrop-2000 UV-Vis Spectrophotometer (Thermo Scientific, Wilmingtoo, DE, USA). The DNA samples were used for both diet (n = 302) and microbiota (n = 300) analyses.<br>The P6 loop of the chloroplast trnL (UAA) region was used for DNA metabarcoding with primers trnL (UAA) g and trnL (UAA) h. For the PCR assays, 10 μL reactions of each of 0.3 μL primers, 0.2 μL KOD FX Neo, 2 μL dNTP, 5 μL KOD FX Neo buffer and 50 ng of DNA template were mixed. Thermocycling followed a program of initial denaturing at 95℃ for 4 min, followed by 35 cycles of 94℃ for 30 s, 50℃for 30 s, and 72℃ for 1 min, with a 5-min final extension at 72℃. All PCRs were conducted with a no-template negative control and a positive control (consisting of DNA extracted from plant species from our local DNA reference library). The 5′ end of each primer was tagged by a 16-nt multiplex identification tag that differed by 8-nt from the other tag, allowing uniquely tagged PCR products. The sequence was carried out on Illumina HiSeq 2500 platform.<br>The V3-V4 region of the 16S rRNA gene was sequenced on Illumina MiSeq 2500 platform with primers (341F/806R). For the PCR assays, 50 μL of each of the 30 ng DNA template, fusion primer and PCR master mix were mixed. The PCR cycles started with a 3 min denaturation at 94℃, followed by 30 cycles each consisting of 94℃ for 30 s, 56℃ for 45 s, 72℃ for 45 s, and followed by a final step of 72℃ for 10 min. PCR products were purified with AmpureXP beads and eluted in elution buffer. Libraries were qualified by the Agilent 2100 bioanalyzer (Agilent, USA). The amplicons were sequenced on Illumina MiSeq 2500 and generated 2×300 bp paired-end reads. Sequence demultiplexing, quality and preliminary identifications were conducted by QIIME 1.9.1. Na Guo, Qunfu Wu and Fuyu Shi performed the procedures and recorded the data step by step.
Timing and spatial scale	Sampling periods spanned the 4 seasons in 2017, namely, spring (May), summer (August), autumn (October) and winter (December) (for transhumance: n=32, spring; n=33, summer; n=37, autumn; n=45, winter and for open-continuous grazing: n=31, spring; n=39, summer; n=38, autumn; n=47, winter).
Data exclusions	No data were exclude.
Reproducibility	The replicates of each samples were done to ensure the reproducibility of experimental findings. All attempts to repeat the experiment were successful.
Randomization	The samples were allocated randomly into groups.
Blinding	NA

Did the study involve field work? ☒ Yes ☐ No

Field work, collection and transport

Field conditions	The annual mean temperature was - 0.1℃ in the transhumance (TH) and 0℃ in the open-continuous grazing (OCG) with a peak in summer (June - August) and a trough in winter (December-February). The annual mean precipitation was 416mm in the TH and 400mm in the OCG. The grassland was alpine meadow and alpine shrub meadow and the plant growing season was 90 - 120 d.
Location	In OCG, the yaks grazed freely on the same area at 3010 to 3300 m above sea level (2000 ha; 37°10′-37°12′N, 102°44′-102°51′E) year-round. In the TH regime, yaks grazed pasture at 2930 to 3000 m above sea level (13.3 ha; 37°12′N, 102°46′E) in winter-spring; at 3130 to 3300 m above sea level (666.7 ha; 37°10′N, 102°44′E) in summer; and at 3015 to 3100 m above sea level (11.3 ha; 37°11′N, 102°44′E) in autumn.

Access & import/export	The studies was done with the permission of local herders. The studies and all procedures involving the animals were approved by the experimental field management protocols (File No: EAF2021012) of Lanzhou University.
Disturbance	As feces were collected, there was no disturbance for animals.

Reporting for specific materials, systems and methods

We require information from authors about some types of materials, experimental systems and methods used in many studies. Here, indicate whether each material, system or method listed is relevant to your study. If you are not sure if a list item applies to your research, read the appropriate section before selecting a response.

Materials & experimental systems

n/a	Involved in the study
☒	☐ Antibodies
☒	☐ Eukaryotic cell lines
☒	☐ Palaeontology and archaeology
☐	☒ Animals and other organisms
☒	☐ Human research participants
☒	☐ Clinical data
☒	☐ Dual use research of concern

Methods

n/a	Involved in the study
☒	☐ ChIP-seq
☒	☐ Flow cytometry
☒	☐ MRI-based neuroimaging

Animals and other organisms

Policy information about studies involving animals; ARRIVE guidelines recommended for reporting animal research

Laboratory animals	NA
Wild animals	Yak (Bos grunniens) was grazed freely on the alpine grassland.
Field-collected samples	The feces were stored at -80 ℃ and used for further analysis (e.g. extracting DNA in the 4 ℃ with ice) in the lab.
Ethics oversight	The studies and all procedures involving the animals were approved by the experimental field management protocols (File No: EAF2021012) of Lanzhou University.

Note that full information on the approval of the study protocol must also be provided in the manuscript.
